# Understanding the biases to sepsis surveillance and quality assurance caused by inaccurate coding in administrative health data

**DOI:** 10.1007/s15010-023-02091-y

**Published:** 2023-09-09

**Authors:** Daniel Schwarzkopf, Norman Rose, Carolin Fleischmann-Struzek, Beate Boden, Heike Dorow, Andreas Edel, Marcus Friedrich, Falk A. Gonnert, Jürgen Götz, Matthias Gründling, Markus Heim, Kirill Holbeck, Ulrich Jaschinski, Christian Koch, Christian Künzer, Khanh Le Ngoc, Simone Lindau, Ngoc B. Mehlmann, Jan Meschede, Patrick Meybohm, Dominique Ouart, Christian Putensen, Michael Sander, Jens-Christian Schewe, Peter Schlattmann, Götz Schmidt, Gerhard Schneider, Claudia Spies, Ferdinand Steinsberger, Kai Zacharowski, Sebastian Zinn, Konrad Reinhart

**Affiliations:** 1 Department of Anesthesiology and Intensive Care Medicine, Jena University Hospital, Am Klinikum 1, 07747 Jena, Germany; 2 Institute of Infectious Diseases and Infection Control, Jena University Hospital, Erlanger Allee 103, 07747 Jena, Germany; 3 Department of Internal Medicine II-Intensive Care, Klinikum Lippe GmbH, Röntgenstraße 18, 32756 Detmold, Germany; 4 Department of Anesthesiology and Operative Intensive Care Medicine (CCM, CVK), Charité-Universitätsmedizin Berlin, corporate member of Freie Universität Berlin and Humboldt-Universität zu Berlin, Augustenburger Platz 1, 13353 Berlin, Germany; 5 Berlin Institute of Health, Visiting Professor for the Stiftung Charité, Anna-Louisa-Karsch-Str. 2, 10178 Berlin, Germany; 6 Department of Anaesthesiology and Intensive Care Medicine, SRH Wald-Klinikum, Straße des Friedens 122, 07548 Gera, Germany; 7 Department of Anaesthesiology, Intensive Care Medicine, Emergency Medicine and Pain Medicine, University Medicine Greifswald, Ferdinand-Sauerbruch-Straße, 17475 Greifswald, Germany; 8 Department of Anesthesiology and Intensive Care Medicine, Technical University of Munich, School of Medicine, Ismaninger Straße 22, 81675 Munich, Germany; 9 Department of Anaesthesiology and Surgical Intensive Care Medicine, Universitätsklinikum Augsburg, Stenglinstr. 2, 86156 Augsburg, Germany; 10 Department of Anesthesiology, Intensive Care Medicine and Pain Therapy, University Hospital Gießen, UKGM, Justus-Liebig University Gießen, Rudolf-Buchheim-Straße 7, 35392 Giessen, Germany; 11 Department of Anaesthesiology, Intensive Care Medicine and Pain Therapy, Goethe University, University Hospital Frankfurt, Theodor-Stern-Kai 7, 60590 Frankfurt am Main, Germany; 12 Department of Anaesthesiology, Intensive Care, Emergency and Pain Medicine, University Hospital Wuerzburg, Oberduerrbacher Straße 6, 97080 Würzburg, Germany; 13 Department of Anaesthesiology and Intensive Care Medicine, University Hospital Bonn, Venusberg-Campus 1, 53127 Bonn, Germany; 14 Department of Anaesthesiology, Intensive Care Medicine, Emergency Medicine and Pain Medicine, University Medical Centre Rostock, Schillingallee 35, 18057 Rostock, Germany; 15 Institute for Medical Statistics, Computer Science and Data Science, Jena University Hospital, Bachstraße 18, 07743 Jena, Germany

**Keywords:** Sepsis, Epidemiology, Quality Assurance, Health Care, Sensitivity and specificity, Administrative Claims, Healthcare

## Abstract

**Purpose:**

Timely and accurate data on the epidemiology of sepsis are essential to inform policy decisions and research priorities. We aimed to investigate the validity of inpatient administrative health data (IAHD) for surveillance and quality assurance of sepsis care.

**Methods:**

We conducted a retrospective validation study in a disproportional stratified random sample of 10,334 inpatient cases of age ≥ 15 years treated in 2015–2017 in ten German hospitals. The accuracy of coding of sepsis and risk factors for mortality in IAHD was assessed compared to reference standard diagnoses obtained by a chart review. Hospital-level risk-adjusted mortality of sepsis as calculated from IAHD information was compared to mortality calculated from chart review information.

**Results:**

ICD-coding of sepsis in IAHD showed high positive predictive value (76.9–85.7% depending on sepsis definition), but low sensitivity (26.8–38%), which led to an underestimation of sepsis incidence (1.4% vs. 3.3% for severe sepsis-1). Not naming sepsis in the chart was strongly associated with under-coding of sepsis. The frequency of correctly naming sepsis and ICD-coding of sepsis varied strongly between hospitals (range of sensitivity of naming: 29–71.7%, of ICD-diagnosis: 10.7–58.5%). Risk-adjusted mortality of sepsis per hospital calculated from coding in IAHD showed no substantial correlation to reference standard risk-adjusted mortality (*r* = 0.09).

**Conclusion:**

Due to the under-coding of sepsis in IAHD, previous epidemiological studies underestimated the burden of sepsis in Germany. There is a large variability between hospitals in accuracy of diagnosing and coding of sepsis. Therefore, IAHD alone is not suited to assess quality of sepsis care.

**Supplementary Information:**

The online version contains supplementary material available at 10.1007/s15010-023-02091-y.

## Introduction

Sepsis is a life-threatening organ dysfunction caused by a dysregulated host response to infection [1]. Recognizing shortcomings in the prevention, diagnosis, and treatment of sepsis, the WHO urged member states to improve epidemiological surveillance as well as the quality of care [2].

Valid information on sepsis incidence and mortality is necessary to inform health policy and clinical research, as well as to benchmark the quality of sepsis care. The majority of studies on the burden of sepsis was based on inpatient administrative health data (IAHD) since these allow easy access to very large databases [3–8]. In these studies, sepsis was identified by International Classification of Diseases (ICD) codes in hospital discharge diagnoses. Surveillance based on administrative data may lead to biased conclusions, if the coding of diagnoses is inaccurate [9]. Several international studies investigated the accuracy of sepsis coding, but most of them had methodological shortcomings, since they included only highly selective samples and did not report all relevant measures of accuracy [10, 11]. Beside our own single-center pilot study, the accuracy of sepsis coding in German IAHD has not yet been studied [12].

Due to their feasibility, administrative data are also used to conduct quality assurance of sepsis care with risk-adjusted mortality being the primary indicator of outcome quality [13–16]. The validity of administrative data for this purpose is currently of especially high interest, since the Federal Joint Committee—the highest authority on quality assurance for German hospitals—is considering introducing mandated quality indicators for sepsis, which will also rely on this kind of data [17]. Quality measures are particularly prone to bias, if the selected population varies between hospitals. However, the variability of accuracy of sepsis coding across hospitals and its consequences for comparing quality of care have not yet been investigated due to a lack of multicenter studies on the topic.

Based on these considerations, we aimed to investigate the accuracy of sepsis coding and its variability across hospitals and to assess the validity of estimates of risk-adjusted mortality from IAHD for measuring quality of sepsis care.

## Material and methods

### Study design

We conducted a multicenter, retrospective, observational validation study. Based on the IAHD of ten German hospitals, a random sample of 10,334 cases treated between 2015 and 2017 was drawn. The validity of coding of sepsis, as well as risk factors in IAHD were then investigated by statistical comparison to reference standard diagnoses obtained via a chart review. The description of the study follows the “Reporting of studies Conducted using Observational Routinely collected health Data” (RECORD) guidelines [18] and the “STAndards for Reporting of Diagnostic accuracy” (STARD) adapted to administrative health data [9]. Passages cited from the published study protocol are not individually marked in the manuscript [19].

### Setting

The study was conducted among a convenience sample of ten hospitals located across Germany recruited within a pre-existing research network (“SepNet”) and a quality collaborative (“German Quality Network Sepsis”). Eight hospitals were university hospitals; two were teaching hospitals providing tertiary-level care. The mean number of beds for inpatient care was 1388 (minimum: 755, maximum: 3000).

### Data sources and study sample

#### Inpatient administrative health data

The study was based on IAHD, which are used for the reimbursement of hospitals in the German diagnosis-related groups (DRG) system. Almost all German hospitals participate in the DRG system. National DRG-statistics can be assessed via the Federal Bureau of Statistics and have been used previously to obtain population estimates of the incidence and mortality of sepsis in Germany [5]. The IAHD contains patient demographics, reasons and type of admission, ICD-10-German-Modification coded diagnoses, conducted surgeries and procedures, treating hospital departments, and discharge destinations (including hospital death).

#### Validation sample

The sample included hospital episodes of patients ≥ 15 years of age, with inpatient somatic treatment from 2015 to 2017. Study centers provided the IAHD in a pseudonymized format. A sample of 1200 hospital episodes per hospital was drawn by disproportional stratified sampling to increase the proportion of “true” sepsis cases in the sample (details provided in the Supplementary Material) [12]. The aim of the study was to review 1000 episodes per hospital; 200 additional episodes were sampled since some medical records might be unavailable. To assure representativeness and avoid bias by learning effects, the review of charts was conducted in random order. The sample size calculation is presented in the Supplement.

### Chart review

Between July 2019 and October 2021, trained study physicians screened all clinical information contained in the medical charts of the validation sample to identify the reference standards. Data were documented in an electronic case report form (eCRF) using the study management software OpenClinica (version 3.1. Copyright © OpenClinica LLC and collaborators, Waltham, MA, USA, www.OpenClinica.com). A training assured the objectivity of the chart review. We assessed interrater agreement between two independent study physicians per study center before the main study based on 40 random cases. Interrater agreement was calculated by Gwet’s AC1, a robust alternative to Cohen’s *κ* [20]. The target value for sufficiently good agreement was set to > 0.6 [21]. Study physicians were blinded on ICD-10-codes in the IAHD, but they could not be blinded to ICD-10 codes in the medical records. Documented medical chart data were linked to IAHD by a study pseudonym (see Supplementary Material for details on training, linkage, and data cleaning).

### Variables

#### Variables derived from chart review

The eCRF of the study was developed based on previous research and a pilot study [10, 12, 22]. The complete CRF is presented in the study protocol [19]. If an infection was present, sepsis was identified both according to the sepsis-1 definition [23, 24], which were the basis for ICD-10-coding of sepsis in Germany until 2019, as well as according to the sepsis-3 definition [1] (see Supplementary Material for details on criteria). For patients with sepsis, clinical characteristics and risk factors for mortality were recorded by trained study nurses. Risk factors were selected based on previous research and included [14]: age, sex, reason for admission (emergency, referral, transfer), comorbidities of the Charlson and Elixhauser indices [25], leukemia, conditions associated with immunosuppression (asplenia, transplanted organ status), and treatments in the current hospital episode associated with increased mortality risk (chemotherapy, stroke treatment).

#### Variables derived from administrative health data

Explicit coding strategies were used to identify patients with infection, sepsis-1 (any ICD-10 sepsis code), sepsis-1 with organ dysfunction (severe sepsis-1 including septic shock, ICD-10 codes R65.1 or R57.2), and septic shock-1 (R57.2). Sepsis-3 and shock according to sepsis-3 were identified by the same codes as severe sepsis-1 (R65.1 or R57.2) and septic shock-1 (R57.2), respectively. Two implicit coding strategies were also investigated to identify cases with severe sepsis-1: a modified Martin definition—the presence of any explicit sepsis code and any ICD-10 code for organ dysfunction [4]—and the Angus definition—the presence of any ICD-10 code for infection and any code for organ dysfunction [3]. Risk factors for sepsis mortality were defined by ICD or OPS (procedures—"Operationen- und Prozedurenschlüssel") codes (definitions of variables provided in the Supplement).

### Statistical methods

Analyses were conducted using the statistical software *R* [26]*.* Survey methods were used to adjust for the clustering of cases in hospitals, sampling weights resulting from the disproportional stratified sampling, and missing values (details provided in the Supplement) [27]. Significance tests were conducted at a bidirectional alpha level of 0.05.

The accuracy of coding of sepsis and risk factors was assessed by sensitivity, specificity, positive predictive value (PPV), and negative predictive value (NPV). Accuracy of sepsis coding was also assessed in the planned subgroups with ICU-treatment and without ICU-treatment.

Since ICD-10 coding of sepsis in the years 2015–2017 still followed sepsis-1 criteria and severe sepsis-1 clinically shows a large overlap to sepsis-3 [28], analyses on the validity of risk-adjusted mortality were based on cases with severe sepsis-1. Three risk models for mortality were calculated by logistic regression: risk model 1 was based on cases with a reference standard diagnosis of severe sepsis-1 and incorporated risk factors identified from chart review (reference model). Risk model 2 used the same cases from the chart review, but incorporated risk factors identified from IAHD. Risk model 3 was based both on sepsis cases and on risk factors identified from IAHD. The influence of miscoding of risk factors on prediction of patient-level risk was investigated by the correlation between comorbidity indices calculated from chart review information vs. comorbidity indices calculated from IAHD information, as well as by the correlation between the individual risk predicted from model 1 vs. the risk predicted from model 2. To assess the influence of miscoding of risk factors and sepsis on hospital-level risk-adjusted mortality, risk-standardized mortality rates (RSMR) were calculated and compared between risk models by scatterplots and by correlations.

To set the results of our study in context with population-level data for Germany, we updated a previously reported analysis of the German national DRG-statistics to the year 2017 (see Supplementary Material for details) [5].

## Results

### Sample

In the training phase, a high interrater agreement was found both for identification of sepsis-1 with organ dysfunction (AC1 = 0.89, 95% CI 0.83, 0.94), as well as sepsis-3 (AC1 = 0.87, 95% CI 0.82, 0.93); the target value of 0.6 was surpassed in all study centers.

A total of 10,334 charts were reviewed in the main study, since some hospitals reviewed more than the required 1000 cases (SFig. 1, Supplementary Material). Sampling weights were adapted accordingly. Chart review identified 3504 cases with infections, which correspond to an incidence of 20.6% (95% CI 18.3%, 23.2%), if sampling weights are adjusted. STable 1 (Supplementary Material) presents descriptive statistics for the individual definition criteria of sepsis. Medical records had missing information to judge the presence of sepsis in 305 cases (3%, sampling weight adjusted) for sepsis-1, and in 764 cases (6.5%) for sepsis-3. Sepsis-1-criteria were fulfilled by 1852 cases (incidence of 6.5% [5.6%, 7.5%] adjusted for sampling weights and missing data), severe sepsis-1 criteria by 1310 (3.3% [2.6%, 4.1%]), sepsis-3 criteria by 1163 (2.9% [1.9%, 4.3%]). Table 1 shows the characteristics of patients with sepsis identified by chart review.

**Table 1:** Characteristics of patients and treatments for cases with sepsis

Variable	Sepsis-1, according to chart review				Sepsis-3, according to chart review		
	*N* missing	Sepsis-1	Severe Sepsis-1	Septic shock-1	*N* missing	Sepsis-3	Septic shock-3
		Statistic	Statistic	Statistic		Statistic	Statistic
*N* in sample		1852	1310	879		1163	653
Proportion of hospital admissions		6.5%	3.3%	1.6%		2.9%	1.1%
Age	0/1852	70 [58, 79]	72 [60, 79]	70 [60, 79]	0/1163	73 [61, 80]	73 [61, 80]
Female sex	0/1852	45.0%	40.8%	44.8%	0/1163	43.0%	43.1%
Type of admission	2/1852				27/1163		
Emergency, surgical		16.2%	17.5%	23.4%		18.4%	25.9%
Emergency, medical		69.6%	69.3%	59.3%		65.3%	56.5%
Planned, surgical		9.7%	10.1%	14.7%		11.8%	14.4%
Planned, medical		4.5%	3.2%	2.7%		4.4%	3.1%
Charlson comorbidity index	259/1852	2 [1, 4]	2 [1, 4]	3 [1, 5]	155/1163	3 [1, 4]	3 [1, 5]
Degree of confirmation of infection	0/1852				0/1163		
Microbiologically proven		54.2%	55.8%	64.8%		59.2%	63.8%
Other confirmation		24.2%	26.5%	21.0%		24.9%	19.3%
Clinically suspected		21.5%	17.7%	14.2%		15.9%	17.0%
Origin of infection	4/1852				3/1163		
Present on admission, nosocomial		10.8%	11.9%	12.1%		11.6%	11.8%
Present on admission, not nosocomial		54.1%	46.3%	43.0%		43.3%	42.6%
Present on admission, unknown origin		7.2%	8.4%	7.6%		8.0%	8.1%
Onset during stay, nosocomial		19.6%	23.0%	25.1%		26.2%	25.9%
Onset during stay, not nosocomial		3.4%	4.4%	5.3%		3.9%	4.1%
Onset during stay, unknown origin		2.3%	2.3%	2.9%		2.5%	3.3%
More than one infection		2.6%	3.6%	4.0%		4.5%	4.1%
Source of infection							
Catheter related infection	146/1852	3.8%	4.2%	6.3%	77/1163	4.6%	7.5%
Central nervous system infection	146/1852	3.1%	2.2%	1.6%	77/1163	2.4%	1.0%
Cardiovascular infection	146/1852	1.2%	2.0%	2.8%	77/1163	2.7%	3.9%
Pneumonia	146/1852	39.1%	56.7%	59.0%	77/1163	55.5%	59.8%
Other respiratory infections	146/1852	9.1%	5.6%	5.4%	77/1163	5.1%	5.9%
Thoracic (empyema/mediastinitis)	146/1852	0.9%	1.6%	2.6%	77/1163	2.0%	2.6%
Intraabdominal infection	146/1852	10.9%	14.6%	19.0%	77/1163	16.1%	22.2%
Gastrointestinal infection	146/1852	13.1%	9.7%	11.0%	77/1163	10.1%	8.7%
Urogenital infection	146/1852	27.5%	22.0%	22.2%	77/1163	24.5%	20.3%
Bones/soft tissue infection	146/1852	10.5%	10.0%	11.8%	77/1163	10.7%	11.6%
Primary bacteremia	146/1852	6.9%	8.0%	7.9%	77/1163	8.4%	8.9%
Other infection	146/1852	2.4%	1.7%	1.0%	77/1163	1.1%	1.1%
Treated on ICU	0/1852	43.6%	65.8%	92.3%	0/1163	74.0%	97.1%
ICU length-of-stay (days)	0/1505	7 [3, 17]	9 [4, 19]	11 [4, 23]	0/1089	9 [4, 21]	11 [4, 23]
Ventilation (including noninvasive)	35/1852				47/1163		
No		66.0%	46.1%	19.8%		38.2%	13.3%
< 24 h		8.9%	9.9%	12.0%		10.5%	12.1%
≥ 24 h		25.1%	44.1%	68.2%		51.3%	74.6%
Extracorporeal membrane oxygenation	4/1852	1.4%	2.4%	4.5%	27/1163	3.0%	5.3%
Renal replacement therapy	7/1852	11.3%	20.2%	33.4%	26/1163	28.3%	41.9%
Liver replacement therapy	2/1852	0.1%	0.1%	0.2%	25/1163	0.2%	0.3%
Vasopressor use	26/1852	30.8%	51.0%	82.9%	45/1163	62.4%	90.6%
Tracheotomy	6/1852	5.9%	10.1%	16.4%	29/1163	12.2%	16.2%
Hospital length-of-stay (days)	0/1852	11 [6, 23]	17 [9, 33]	21 [9, 37]	0/1163	19 [9, 34]	20 [8, 35]

Descriptive statistics given as relative frequencies (%) or median [1^st^ quartile, 3^rd^ quartile]. Absolute frequencies are given for the sample, and all descriptive statistics (relative frequency and median) are calculated with adjustment for sampling weights and clustering

### Accuracy of sepsis coding

The accuracy of identifying infection and sepsis by explicit ICD-10-codes is presented in Table 2. In general, explicit coding identified sepsis with high specificity (≥ 99.5%), but low sensitivity (≤ 38%). Only 34.4% (21.6%, 49.9%) of cases identified as showing severe sepsis-1 from chart review also had a respective ICD-10 code in IAHD (sensitivity). Among cases with an explicit code for severe sepsis-1, 83.3% (71.6%, 90.8%) had severe sepsis-1 according to chart review (PPV). There were no substantial changes in accuracy of coding from 2015 to 2017 (Supplementary Material—STable 2). For ICU-treated cases, accuracy of coding was better compared to cases without ICU-treatment. Identifying severe sepsis-1 by ICD-codes resulted in estimating the incidence with 1.4% (0.8%, 2.3%) compared to 3.3% (2.6%, 4.1%) estimated from chart reviews (underestimation by factor 2.35). At the same time, hospital mortality of explicitly coded cases was overestimated (41.9% [29.1%, 55.9%] compared to 27.8% [21%, 35.8%]). In general, explicit coding of sepsis was associated with an underestimation of incidence and an overestimation of mortality (Table 2). Implicit coding strategies for identification of severe sepsis-1 did not result in improved accuracy (Supplementary Material—STable 3), but only increased sensitivity (modified Martin definition: 40.5% [30.3%, 51.5%], Angus definition: 72.7% [63.8%, 80.1%], respectively) at the cost of decreasing PPV (74.0% [61.2%, 83.7%] and 35.0% [28.0%, 42.7%], respectively).

**Table 2:** Accuracy of identification of cases with infection or sepsis by explicit ICD-10-codes

	Explicit coding of infection or sepsis		Reference standard from chart review			Predictive accuracy of explicit coding of infection or sepsis			
	Proportion of hospital admissions	Hospital mortality	*N* missing	Proportion of hospital admissions	Hospital mortality	Sensitivity	Specificity	PPV	NPV
All cases									
Infection	23.7% [22%; 25.4%]	6% [4.5%; 8.1%]	0/10,384	20.6% [18.3%; 23.2%]	6.7% [5%; 9%]	79.1% [71.8%; 84.9%]	90.7% [89.2%; 92%]	68.9% [63.6%; 73.8%]	94.4% [91.8%; 96.2%]
Sepsis-1	2.3% [1.8%; 3%]	28% [20.9%; 36.3%]	185/10,384	6.5% [5.6%; 7.5%]	16.2% [11.6%; 22.2%]	27.5% [19.7%; 36.9%]	99.5% [99.1%; 99.7%]	78.3% [65.4%; 87.3%]	95.2% [94.1%; 96%]
Severe sepsis-1	1.4% [0.8%; 2.3%]	41.9% [29.1%; 55.9%]	275/10,384	3.3% [2.6%; 4.1%]	27.8% [21%; 35.8%]	34.4% [21.6%; 49.9%]	99.8% [99.6%; 99.8%]	83.3% [71.6%; 90.8%]	97.8% [97.4%; 98.2%]
Septic shock-1	0.5% [0.3%; 0.9%]	61% [46.6%; 73.8%]	305/10,384	1.6% [1.3%; 2%]	44.9% [38.5%; 51.4%]	26.8% [14.9%; 43.3%]	99.9% [99.8%; 100%]	85.7% [61.2%; 95.8%]	98.8% [98.5%; 99.1%]
Sepsis-3	1.4% [0.8%; 2.3%]	41.9% [29.1%; 55.9%]	658/10,384	2.9% [1.9%; 4.3%]	31.2% [22.7%; 41.1%]	38.0% [28.6%, 48.5%]	99.8% [99.6%, 99.9%]	84.5% [69.2%, 93.0%]	98.2% [97.5%, 98.7%]
Septic shock-3	0.5% [0.3%; 0.9%]	61% [46.6%; 73.8%]	439/10,384	1.1% [0.8%; 1.5%]	54.4% [47.6%; 60.9%]	35.7% [19.1%, 56.6%]	99.9% [99.8%, 99.9%]	76.9% [65.0%, 85.7%]	99.3% [98.9%, 99.5%]
No ICU-treatment									
Infection	21.0% [19.4%, 22.7%]	2.6% [1.1%, 6.0%]	0/5565	17.6% [15.0%, 20.5%]	2.8% [1.2%, 6.3%]	77.5% [69.1%; 84.1%]	91.1% [89.7%; 92.3%]	64.9% [59.1%; 70.4%]	95.0% [92.3%; 96.8%]
Sepsis-1	1.1% [0.8%, 1.6%]	12.8% [3.3%, 38.8%]	114/5565	4.2% [3.3%, 5.1%]	7.5% [2.6%, 20.0%]	17.4% [9.9%; 28.7%]	99.6% [99.2%; 99.8%]	63.9% [43.5%; 80.2%]	96.5% [95.4%; 97.4%]
Severe sepsis-1	0.4% [0.2%; 1.1%]	23.4% [4.1%; 68.4%]	166/5565	1.3% [0.9%; 1.7%]	14.6% [4.9%; 36.3%]	24.8% [9.4%; 51.1%]	99.9% [99.8%; 99.9%]	70.5% [50.4%; 84.9%]	99.0% [98.7%; 99.3%]
Septic shock	0% [0%; 0.1%]	50.4% [15.4%; 85%]	179/5565	0.1% [0.1%; 0.3%]	37.6% [10.3%; 76%]	3.0% [0.4%; 18.8%]	100.0% [99.9%; 100.0%]	18.4% [0.1%; 98.9%]	99.9% [99.7%; 99.9%]
Sepsis-3	0.4% [0.2%; 1.1%]	23.4% [4.1%; 68.4%]	390/5565	0.9% [0.5%; 1.6%]	13.7% [3.4%; 41.3%]	28.6% [14.8%, 48.0%]	99.9% [99.7%, 99.9%]	68.7% [35.7%, 89.6%]	99.4% [99.1%, 99.6%]
Septic shock-3	0% [0%; 0.1%]	50.4% [15.4%; 85%]	306/5565	0% [0%; 0.2%]	48.2% [6.4%; 92.7%]	10.4% [2.8%, 32.2%]	100.0% [99.8%, 100.0%]	14.8% [0.1%, 98.3%]	100.0% [99.8%, 100.0%]
ICU-treatment									
Infection	44.9% [42.1%, 47.8%]	18.5% [16.1%, 21.0%]	0/4819	44.5% [40.9%, 48.2%]	19.1% [16.0%, 22.6%]	84.3% [79.3%; 88.3%]	86.6% [81.4%; 90.5%]	83.5% [76.2%; 88.8%]	87.3% [83.6%; 90.3%]
Sepsis-1	11.3% [9.1%, 13.9%]	40.1% [33.7%, 47.0%]	71/4819	25.0% [20.0%, 30.8%]	27.6% [24.0%, 31.5%]	40.4% [34.1%; 47.2%]	98.4% [96.9%; 99.2%]	89.4% [77.5%; 95.4%]	83.2% [78.5%; 87.1%]
Severe sepsis-1	8.4% [5.8%; 12%]	49.7% [42.8%; 56.6%]	109/4819	18.8% [14.7%; 23.8%]	34.7% [29%; 40.9%]	39.4% [28.6%; 51.4%]	98.8% [97.6%; 99.4%]	88.6% [74.8%; 95.3%]	87.5% [83.8%; 90.5%]
Septic shock-1	4.2% [2.4%; 7.3%]	61.9% [49.4%; 73.1%]	126/4819	12.9% [9.9%; 16.7%]	45.5% [39.5%; 51.6%]	28.7% [16.2%; 45.7%]	99.4% [98.6%; 99.8%]	88.6% [70.1%; 96.3%]	90.4% [87.2%; 92.8%]
Sepsis-3	8.4% [5.8%; 12%]	49.7% [42.8%; 56.6%]	268/4819	18.5% [12.6%; 26.3%]	37.4% [30%; 45.3%]	41.3% [32.4%, 50.7%]	98.9% [97.9%, 99.5%]	89.7% [76.6%, 95.9%]	88.1% [82.5%, 92.1%]
Septic shock-3	4.2% [2.4%; 7.3%]	61.9% [49.4%; 73.1%]	133/4819	9.5% [6.9%; 12.9%]	54.3% [48%; 60.5%]	36.1% [19.7%, 56.6%]	99.0% [98.3%, 99.5%]	79.6% [74.2%, 84.1%]	93.7% [90.2%, 96.0%]

Missing values on the reference standard result from lacking information on criteria of sepsis in medical records, absolute frequencies give numbers for the sample. Estimates of proportion of infection or sepsis cases, hospital mortality, sensitivity, specificity, PPV (positive predictive value), and NPV (negative predictive value) are adjusted for sampling weights and clustering and are reported with their 95% confidence intervals (CI)

**Fig. 1:**
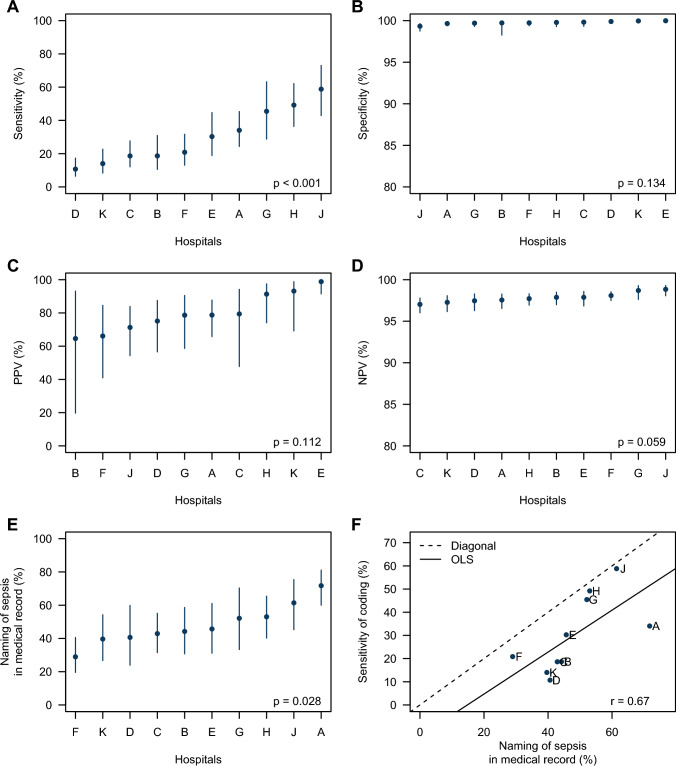
Accuracy of explicit coding of severe sepsis-1 in inpatient administrative health data. All estimates are adjusted for sampling weights and clustering. Explicit coding of severe sepsis-1 is defined by the presence of ICD-10 codes R65.1 or R57.2 in inpatient administrative health data (IAHD). Whiskers in panels **a–e** present 95% confidence intervals. p values in panels **a**–**e** obtained by Rao–Scott Pearson *χ*^*2*^-test with Satterthwaite approximation. Panel **a**: sensitivity of coding of severe sepsis-1 per hospital. Panel **b**: specificity of coding of severe sepsis-1 per hospital. Panel **c**: positive predictive value (PPV) of coding of severe sepsis-1 per hospital. Panel **d**: negative predictive value (NPV) of coding of severe sepsis-1 per hospital. Panel **e**: frequency of naming “sepsis” in the chart among cases with severe sepsis-1 according to chart review. Panel **f**: scatterplot of frequency of naming of sepsis and sensitivity of coding of severe sepsis-1; OLS is the ordinary least squares approximation line; *r* is the Pearson correlation

Participating hospitals showed large differences in the accuracy of sepsis coding (Fig. 1a–d). The sensitivity of coding of severe sepsis-1 ranged between 10.7% and 58.5% (median: 25.6%, 1st quartile: 18.6%, 3rd quartile: 42.6%; test of difference: *p* < 0.001, Fig. 1a); the PPV ranged between 64.6% and 98.8% (median: 78.7%, 1st quartile: 72.3%, 3rd quartile: 88.3%; *p* = 0.112, Fig. 1b). Variability of accuracy was comparably large for the other explicit coding strategies (Supplementary Material—SFig. 2).

Among cases with a reference standard diagnosis of severe sepsis-1, sepsis was only named in 44% (36.4%, 51.9%) of discharge letters and in 49.7% (42.6%, 56.8%) of medical records (Table 3). Among cases without naming of sepsis, the probability of a true positive coding was 7.6% (3.3%, 16.7%). Naming of sepsis in the chart increased the probability of correct coding to 61.8% (41.2%, 78.9%). This means that 38.2% of true sepsis cases were not ICD-coded although they had been documented by treating clinicians. There was a large variability of naming sepsis between hospitals (range 29–71.7%, median: 45%, 1st quartile: 41.2%, 3rd quartile: 52.8%; *p* = 0.028; Fig. 1e). Hospitals with a higher frequency of naming sepsis also showed a higher frequency of ICD-coding of sepsis (correlation of *r* = 0.67, Fig. 1f).

**Table 3:** Naming of sepsis in medical record of patient with sepsis according to sepsis-1 definitions

Variable	Reference standard diagnosis of sepsis-1 by chart review		
	Sepsis-1	Severe sepsis-1 (including septic shock)	Septic shock
Sepsis diagnosis named in the medical record (including discharge letter)	31.8% [24.4%, 40.1%]	49.7% [42.6%, 56.8%]	64.3% [54.9%, 72.7%]
Sepsis diagnosis named in the medical record (excluding discharge letter)	25.9% [19.2%, 33.9%]	41.3% [34.1%, 49.0%]	58.2% [48.4%, 67.5%]
Sepsis diagnosis named in discharge letter	28.0% [21.0%, 36.2%]	44.0% [36.4%, 51.9%]	56.3% [45.8%, 66.3%]
Type of sepsis diagnosis named in the discharge letter			
Sepsis without organ dysfunction	12.4% [6.5%, 22.4%]	7.7% [2.6%, 20.9%]	1.5% [0.8%, 2.8%]
Severe sepsis (but not septic shock)	15.4% [7.9%, 27.7%]	18.7% [10.2%, 31.7%]	14.4% [7.5%, 26.1%]
Septic shock	30.2% [22.7%, 39.1%]	38.4% [26.1%, 52.4%]	58.0% [44.9%, 70.1%]
Type not classified	41.9% [28.0%, 57.3%]	35.2% [23.4%, 49.2%]	26.1% [16.9%, 37.9%]

Estimates are presented as relative frequencies (%) along with their 95% confidence intervals and were calculated with adjustment for sampling weights and clustering

### Validity of risk-adjusted mortality estimated from IAHD

**Fig. 2:**
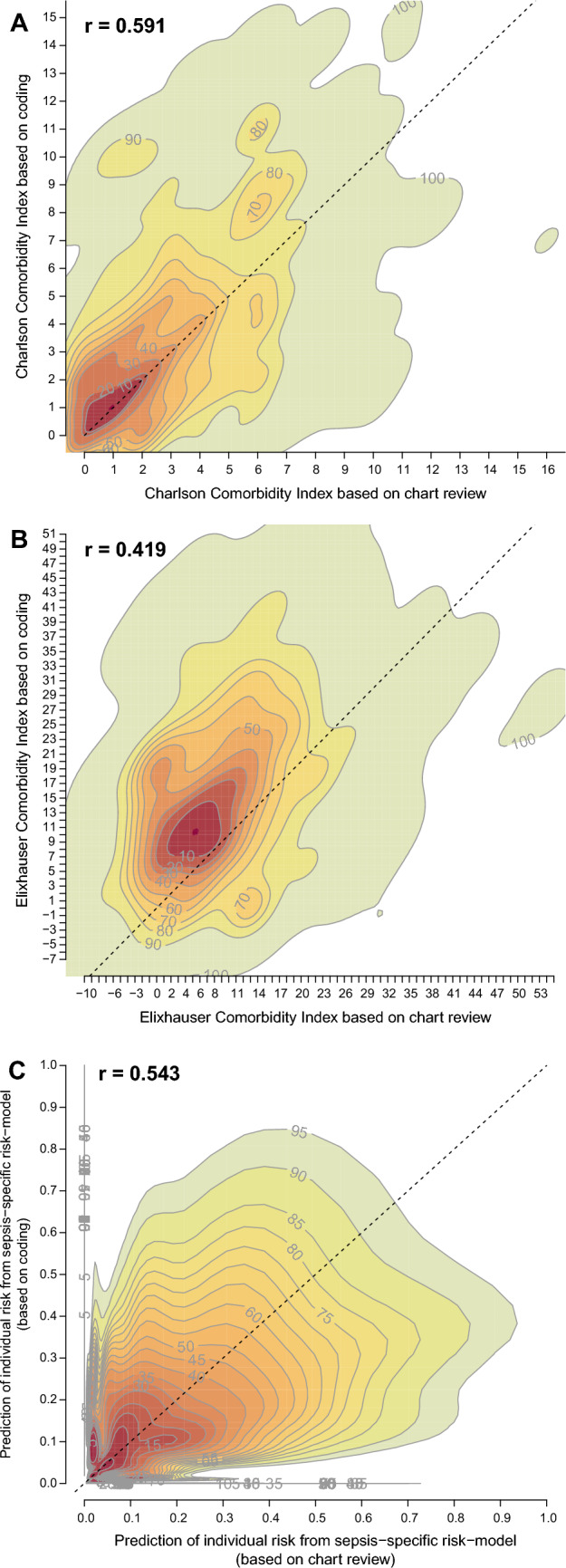
Prediction of individual risk of death during the hospital stay comparing information based on administrative health data with information from chart review. Presented are contour plots; *r* presents the Pearson correlation. Analyses are based on the sample of cases with severe sepsis-1 according to chart review. Figures and correlations are adjusted for sampling weights and clustering. Panel **a**: contour plot of Charlson Comorbidity Index calculated from comorbidities as identified by chart review (*X*-axis) compared to information obtained from inpatient administrative health data (IAHD, *Y*-axis). Panel **b**: contour plot of Elixhauser Comorbidity Index calculated from comorbidities as identified by chart review (*X*-axis) compared to information obtained from IAHD (*Y*-axis). Panel **c**: contour plot of individual risk of death predicted from the sepsis-specific risk model 1 including risk factors identified by chart review (*X*-axis) and from the model 2 including risk factors identified from IAHD (*Y*-axis)

**Fig. 3:**
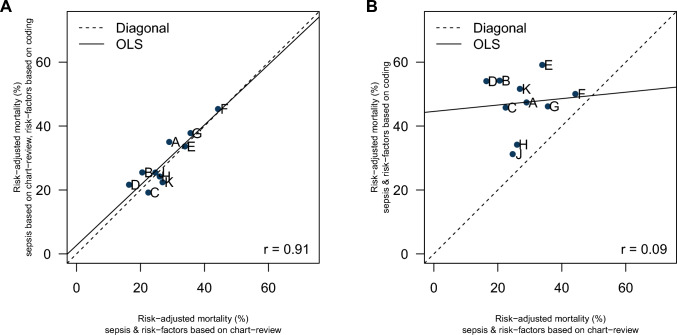
Risk-adjusted hospital mortality of patients with severe sepsis-1 comparing information from coding in administrative health data with information from chart review. All estimates of risk-adjusted mortality (dots) are adjusted for sampling weights and clustering. Individual hospitals are represented by capital letters. Panel **a**: scatterplot of risk-standardized mortality rates (RSMRs) calculated from a model based on cases with severe sepsis-1 as well as risk factors identified in chart review (reference model 1, *X*-axis) and RSMRs calculated from a model based on cases with severe sepsis-1 identified in chart review but risk factors identified by coding in inpatient administrative health data (model 2, *Y*-axis). Panel **b**: scatterplot of RSMRs calculated from a model based on cases with severe sepsis-1 as well as risk factors identified in chart review (reference model 1, *X*-axis) and RSMRs calculated from a model completely based on coding in inpatient administrative health data (identification of cases with severe sepsis-1 as well as risk factors, model 3, *Y*-axis)

The accuracy of coding of risk factors was low in general, with a large variation across the different risk factors—ranging from a sensitivity of 0.9% for peptic ulcer disease to 96.2% for previous solid organ transplantation (median: 53.2%, 1st quartile: 36.5%, 3rd quartile: 75.1%; Supplementary Material—STable 4). The Charlson Comorbidity Index, when calculated from coded information, showed only a mediocre correlation to the index calculated from chart review information (*r* = 0.59, Fig. 2a); same was true for the Elixhauser Comorbidity Index (*r* = 0.42, Fig. 2b) and for the individual risk of death calculated from the sepsis-specific risk models 1 and 2 (*r* = 0.54; Fig. 2c). If risk-adjusted hospital mortality per hospital was calculated based on reference standard sepsis cases but with risk factors from coding in IAHD, it showed a high correlation of *r* = 0.91 to risk-adjusted mortality, where both sepsis and risk factors were based on reference standard information (Fig. 3a). If in addition the calculation of risk-adjusted mortality was based on sepsis cases as coded in IAHD the correlation to reference standard risk-adjusted mortality was essentially cero (*r* = 0.09, Fig. 3b).

### Sepsis cases coded in the national German DRG-statistics

We identified 148,288 hospitalized cases with ICD-codes for severe sepsis-1 including septic shock—corresponding to 0.87% of hospitalizations and 207 cases per 100,000 inhabitants ≥ 15 years, of which 59,792 (40.3%) died.

## Discussion

This study investigated the validity of IAHD for epidemiologic surveillance and quality management of sepsis by comparing information from IAHD to a reference standard obtained by a chart review in ten German hospitals. It showed that the accuracy of identification of sepsis cases based on ICD-10-codes in IAHD was low, leading to an under-coding of sepsis. There was a large variability of coding accuracy across the ten participating hospitals. Under-coding of sepsis was strongly related to lacking documentation of a sepsis diagnosis in the clinical record. Sepsis-related risk-adjusted hospital mortality estimated from IAHD showed no correlation to the risk-adjusted mortality from the chart review, which makes it currently unsuitable to assess outcome quality of sepsis care based on IAHD.

Only a few studies reported estimates of PPV, NPV, sensitivity, and specificity for coding of sepsis in a general sample of hospitalized patients [10, 11]. They observed sensitivity between 9 and 47% and PPV between 75 and 100% for explicit sepsis coding compared to a reference standard for severe sepsis-1 or sepsis-3 [22, 29–31]. These results correspond to our finding that while the majority of “true” sepsis cases are not explicitly coded in IAHD, the majority of coded cases “truly” have sepsis. In consequence, we observed an underrepresentation of sepsis in IAHD. Based on explicit ICD-codes for severe sepsis-1 in the national German DRG-statistics, an incidence of 207 cases per 100,000 inhabitants above 14 years of age was estimated. If the relative frequency of sepsis cases among hospitalizations of 3.3%—as found by chart review in our study—would apply to the German population, this would instead result in an incidence of 785/100,000. This number is in the same range, as those, which have been inferred from representative studies in other countries. For example, an incidence of 687/100,000 and 780/100,000 for severe sepsis-1 and sepsis-3, respectively, was found in Sweden by a chart review study [32]. Likewise, based on identification of sepsis cases in electronic health records of 409 hospitals, a total of 1.7 million adult sepsis cases have been estimated for the USA, which corresponds to an incidence of 710/100,000 (sepsis-3) [29]. The respective mortality rates for sepsis-3 in these studies were 17.4% (Sweden) and 23.2% (USA). The hospital mortality rate of 31.2% for sepsis-3 derived from the chart review in the current study was considerably higher, possibly indicating potential for improvement of care in Germany.

Like a previous validation study, we found that mortality was higher among coded sepsis cases compared to reference standard cases [33]. Therefore, increases of incidence of ICD-coded sepsis across time—for example by awareness campaigns, improved screening protocols, or financial incentives—could be accompanied by a reduction in mortality because of recognition of less severely ill patients. Numerous international studies based on administrative data have reported this pattern across time [4, 5, 34], which is most likely to a large part caused by a methodological bias [34]. Likewise, Rhee et al. replicated the described pattern based on ICD-coded sepsis in their representative US study, but also found incidence and mortality of sepsis as estimated from electronic health record data to be much more stable [29]. Therefore, administrative data alone are not suited for sepsis surveillance across time or for comparing sepsis incidence and mortality between different health care systems [5, 34].

Comparable to results of a small single-center study in the USA [33], we found that the lacking naming of sepsis in the medical record was strongly correlated with under-coding of sepsis in IAHD. It is alarming that among patients with severe sepsis-1 only half of the medical records contained the word “sepsis”, which was likely also associated with inadequate treatment [35]. There was a large variation in the naming of sepsis between hospitals, indicating large differences in sepsis awareness. This highlights the importance of making training of all medical staff on signs and symptoms of sepsis obligatory for all German hospitals, as is intended by the current proposal for a mandated sepsis quality indicator [17]. Even among correctly named sepsis cases, only about 60% received a respective ICD-code for sepsis. The likely explanation is that a sepsis diagnosis often does not increase the reimbursement for German hospitals. Therefore, to address the problem of under-coding, the rules and incentives for ICD-coding of sepsis need to be changed too.

We found the accuracy of the coding of risk factors for sepsis-related mortality to be low. Consequently, comorbidity indices calculated from IAHD were only modestly correlated with the same indices from reference standard information. Although similar findings have been previously reported for other patient populations [36, 37], ICD-based comorbidity scores are still widely used in research—usually ignoring potential biases due to unreliable coding [38]. Nonetheless, the major problem in using IAHD for assessment of quality of sepsis care is not the inaccurate coding of comorbidity but the inaccurate coding of sepsis itself. When only risk factors were defined based on imperfect coding information but valid sepsis cases from chart review were used, the correlation to the reference standard model was high. This corresponds to previous studies, which showed that inaccurately coded risk factor caused only limited bias to hospital benchmarks [13, 39]. When in addition, also, sepsis cases were identified based on ICD-coding, there was virtually no correlation left to the reference standard. This is due to the large variability in accuracy of coding across the participating hospitals. The large variation in the accuracy of administrative data for identifying sepsis across hospitals and the resultant inconsistency in benchmarks of mortality have previously also been shown in comparison with sepsis cases identified by an algorithm in electronic health record data [40]. The sensitivity of coding is itself influenced by sepsis awareness, which is a primary target for quality initiatives. In consequence, administrative data in their current form cannot provide a firm ground to benchmark the outcome quality of sepsis care. This is a great challenge for the planned mandated sepsis quality indicator for German hospitals [17].

### Strengths and limitations

This is the first study to investigate the variability of accuracy of sepsis coding across hospitals, which was therefore able to draw conclusions on the validity to measure quality of care for sepsis based on administrative data. The study used an unselected sample of all cases treated in the included hospitals, provided all relevant measures of coding accuracy and used a rigorous review of medical records to define the reference standard—thereby surpassing methodological shortcomings of most previous studies on the topic [10, 11]. The study is limited by using a small convenient sample of university and tertiary care hospitals, which impairs the generalization of our results to the German population and biases comparisons with epidemiological numbers from other countries. Missing data prevented from assessing the reference standard in all sampled cases, but were handled by adequate techniques from survey research. Although the German ICD-10 did not implement sepsis-3 definitions until 2020, the publication of sepsis-3 in 2016 might have already influenced diagnostic and documentation by clinicians during our study period. We therefore focused on severe sepsis-1, which in clinical practice largely overlaps with sepsis-3 [28] and found no substantial changes in accuracy of coding between 2015 and 2017. Our results need replication to reflect current coding practices after the complete implementation of sepsis-3-definitions. To better understand differences in coding practices between hospitals, future studies should involve the responsible medical coders to conduct in depth analysis of coding decisions.

## Conclusions

Administrative health data in their current form are not valid to identify cases with sepsis or risk factors for sepsis-related mortality. Since sepsis is under-coded, previous epidemiological studies, which were based on administrative health data, severely underestimated incidence, as well as burden in deaths, morbidity, and cost related to sepsis in Germany [5, 41]. Because of the large variation in sepsis awareness and validity of coding across hospitals, administrative data in their current form are not suited for benchmarking quality of sepsis care. Since prospective inclusion of cases with sepsis or retrospective chart review is too burdensome to implement continuous surveillance and quality management [6], newer ways to overcome shortcomings of administrative data need to be found. Implicit strategies have been proposed to improve the identification of sepsis cases in IAHD [10, 30]; but like other studies before, our study showed that implicit strategies only increased sensitivity at the cost of PPV [12, 22, 29, 31]. Another approach is natural language processing. This approach can help to identify sepsis cases in medical records, but has limited ability to solve the problem of lacking awareness and documentation by treating clinicians [42]. Most promising are probably algorithms, which use electronic health record data to combine information indicating the presence of infection with information indicating the presence of organ dysfunction to identify sepsis. These achieved higher sensitivity compared to explicit ICD-coding of sepsis [29], but a recent study indicated that this might also come at the cost of reduced specificity and PPV [43]. Such algorithms might be less prone to differences in diagnosis and documentation of sepsis [40], but the variation of their precision across hospitals still needs to be investigated. The lacking adoption and standardization of electronic health records in Germany currently hinders further progress in this direction and therefore needs to be addressed by the responsible regulatory bodies. Finally, training in awareness, adequate clinical documentation, and ICD-coding of sepsis could improve both sepsis care as well as the validity of administrative data for surveillance and quality assessment [44]. Influenced by discussions of our results, new ICD-10 codes have been introduced in Germany in 2023 to allow a better representation of sepsis [45]. To aid clinicians and medical coders, the German Quality Network Sepsis and the German Sepsis Society recently issued a guideline for sepsis documentation and coding [46].

### Supplementary Information

Below is the link to the electronic supplementary material.

Supplementary file1 (PDF 826 KB)

## Data Availability

The study protocol has been published with open access (https://bmjopen.bmj.com/content/10/10/e035763.info). The statistical analysis plan is available from the study’s registration page (https://drks.de/search/de/trial/DRKS00017775). Deidentified participant data are available from the corresponding author on reasonable request (E-Mail: Daniel.Schwarzkopf@med.uni-jena.de). Access to anonymized data might be granted following review and permission of a study proposal by the ethics commission and data protection officer of the Jena University Hospital, as well as by the involved study centers.
