# Supplementary material for: Understanding the biases to sepsis surveillance and quality assurance caused by inaccurate coding in administrative health data
Source: Infection. 2023 Sep 9;52(2):413–27. doi: 10.1007/s15010-023-02091-y (PMC10954942; doi:10.1007/s15010-023-02091-y)
Supplement: Supplementary file 1 — Supplementary file1 (PDF 826 KB) [file 15010_2023_2091_MOESM1_ESM.pdf]

Supplementary Material

Article title: Understanding the biases to sepsis surveillance and quality assurance caused by inaccurate coding in administrative health data

Authors: Daniel Schwarzkopf, Norman Rose, Carolin Fleischmann-Struzek, Beate Boden, Heike Dorow, Andreas Edel, Marcus Friedrich, Falk A. Gonnert, Jürgen Götz, Matthias Gründling, Markus Heim, Kirill Holbeck, Ulrich Jaschinski, Christian Koch, Christian Künzer, Khanh Le Ngoc, Simone Lindau, Ngoc B. Mehlmann, Jan Meschede, Patrick Meybohm, Dominique Ouart, Christian Putensen, Michael Sander, Jens-Christian Schewe, Peter Schlattmann, Götz Schmidt, Gerhard Schneider, Claudia Spies, Ferdinand Steinsberger, Kai Zacharowski, Sebastian Zinn, Konrad Reinhart

Corresponding author: Daniel Schwarzkopf; Department of Anesthesiology and Intensive Care Medicine, Jena University Hospital, Jena, Germany; E-Mail: Daniel.Schwarzkopf@med.uni-jena.de

Content

1 Supplemental Methods ... 3
1.1 Sampling strategy ... 3
1.2 Sample size calculation ... 3
1.3 Linkage ... 3
1.4 Training and assessment of interrater agreement ... 3
1.5 Data cleaning ... 3
1.6 Statistical analysis using survey methods ... 4
2 Definition of sepsis for the chart review ... 5
2.1 Definition of infection ... 5
2.2 Definition of systemic inflammatory response syndrome (SIRS) according to sepsis-1 definition ... 5
2.3 Presence of organ dysfunction according to sepsis-1 definition ... 6
2.4 Presence of organ dysfunction according to sepsis-3 definition ... 9
2.5 Presence of septic shock according to sepsis-3 definition ... 10
3 Definition of variables in administrative health data ... 11
3.1 Explanation ... 11
3.2 Variable derived by recoding of variables in data table „FALL“ ... 11
3.3 Variables based on ICD-codes ... 11
3.4 Variables based on OPS-codes ... 14
3.5 Variables calculated using previously defined variables. ... 15
4 Analysis of the German DRG-statistics ... 16
4.1 Background ... 16
4.2 Design ... 16
4.3 Setting ... 16
4.4 Sample ... 16
4.5 Procedure ... 16
4.6 Statistical analysis ... 16
4.7 Results ... 16
5 Supplemental Figures ... 17
6 Supplemental Tables ... 19
7 Supplemental references ... 23

1 Supplemental Methods

1.1 Sampling strategy

A sample of 1,200 hospital episodes per hospital was drawn by the study's epidemiologist. Since the rate of sepsis among hospital cases was estimated at only about 2% [1-3], a disproportional stratified sampling was done to increase the proportion of "true" sepsis cases in the sample. The strata were defined by the cross-tabulation of the following criteria: a) presence of a procedure code (*Operationen- und Prozedurenschlüssel*; OPS) for complex intensive care treatment (yes vs. no, OPS-code: 8-890); b) hospital length of stay (≤ 6 days vs. > 6 days), c) year of discharge (2015 to 2017). The same number of cases was sampled from each of the resulting 12 strata. The strata were chosen based on the experiences from a single-centre pilot study, where the rate of cases with sepsis-1 in a sample obtained by this method was 16% [2].

1.2 Sample size calculation

The sample size was calculated regarding the primary endpoint of the study – the sensitivity of the coding of sepsis-1 with organ dysfunction (ICD-10-GM codes R65.1 and R57.2) in IAHD. In the pilot study the sensitivity was estimated to be 0.39 [2]. To estimate sensitivity with a 95% confidence interval of width ±0.03 a sample of number of about 850 true sepsis cases with organ dysfunction according to judgement of record data are necessary. In our pilot study, the rate of such cases in the sample was 8.6% resulting in necessary total sample of number of about 10,000 hospital episodes.

1.3 Linkage

IAHD were pseudonymized within the participating hospitals; hospitals kept a list linking internal case identification numbers with the pseudonym to identify the randomly selected charts. To assure correct linkage patients' age, gender, exact time of admission and discharge obtained from IAHD were provided in the list of selected cases. The pseudonym was used to identify the cases of the validation sample within the eCRF and thereby to link the data from medical records with the information obtained from the IAHD. The linkage was conducted by the study's epidemiologist at the Jena University Hospital. The quality of the linkage was evaluated by comparing demographic information between the IAHD and the eCRF-data.

1.4 Training and assessment of interrater agreement

Cases with sepsis in the validation sample were identified by trained study physicians in a chart review conducted in the respective study centre. Study physicians worked in the respective hospitals and were either examined intensivist or supervised by an examined intensivist. For purpose of training of study physicians to identify cases with sepsis from medical records, 40 cases were sampled per study centre including 20 cases with coded sepsis with organ dysfunction or septic shock (ICD-10-GM codes R65.1 or R57.2), 10 cases with coding of any other infection, and 10 cases without any infection code. Based on a written working instruction and a training session with the coordinating study physician, all local study physicians (at least two necessary) of the centres reviewed and discussed every of the 40 cases, of which five were monitored by the coordinating study physician. After the training, a second sample of 40 cases was provided to assess the objectivity of the chart review process. These cases were reviewed independently by two trained study physicians and information on sepsis criteria was documented in an eCRF. Interrater agreement was calculated by Gwet's AC1, a robust alternative to Cohen's κ [4]. The target value for sufficiently good agreement was set to > 0.6 [5]. A high agreement was found both for identification of sepsis-1 with organ dysfunction (*AC1* = 0.89, 95% CI: 0.83 - 0.94), as well as sepsis-3 (*AC1* = 0.87, 95% CI: 0.82 – 0.93); the target value was surpassed in all study centers.

1.5 Data cleaning

Since the IAHD are used for billing, they are highly standardized and hospitals invest efforts to guarantee the correctness of the documented data. Pseudonymization was done within the participating hospitals using the 3M^{TM} Cryptowizard – a standalone software, which allows a user-friendly pseudonymization of the IAHD in a point-and-click interface.

The eCRF included several methods to foster correct documentation of data: most items included a separate category to indicate missing information ("unknown"); conditional rules were used to implement nested items, a data manager checked the completeness of documentation and managed queries together with the local study nurses. To guarantee correct documentation of sepsis criteria, an active feedback was implemented in the eCRF: after the criteria were documented by the study physician, the eCRF presented, which sepsis categories would apply to the case based on the documentation. The study physician then had to actively confirm this categorization or could correct the documentation of sepsis criteria if any inconsistencies were apparent. The

technical aspects of the eCRF were extensively pre-tested before the main study was conducted. Cleaning and data preparation were conducted by the study's epidemiologist; "unknown" categories of variables were set to missing values.

1.6 Statistical analysis using survey methods

The R-package *survey* was used to calculate relative frequencies and logistic regressions for complex data [6]. Missing values due to lacking information in medical records were treated by missing-data adjusted sampling weights to prevent bias by over- or underrepresentation of strata [7]. Classification trees were calculated using the R-package *rpart*, which also allows to take sampling weights into account [8]. Weighted correlations between variables obtained from chart review with variables obtained from IAHD (comorbidity indices, predicted risk from risk-models) were calculated using the R package *jtools* [9]. The bivariate relations between these variables were visualized by contour plots, which were created using two-dimensional weighted kernel-density estimates via the package *ks* [10].

2 Definition of sepsis for the chart review

In the following, we present the CRF items for the documentation of sepsis during the chart review as well as the working instruction. The complete CRF has been published along with the study protocol [11]. CRF items are given in sans serif font with red colour text presenting a filter criterion and blue colour text presenting a multiple-choice option.

2.1 Definition of infection

1. Presence of infection during hospitalisation?

1 a. Presence of infection	☐ 0 no<br>☐ 1 yes

Document if an infection was present in the reviewed case. Your decision should be based on all available data including the electronic as well as paper record.

1 b. *if 1a = yes,* highest degree of confirmation?	☐ 1 microbiologically proven<br>☐ 2 other confirmation of infection (i.e. radiological finding with according clinical syndrome, conspicuous urine status)<br>☐ 3 clinically suspected (increased infection levels, fever)

Document the degree of confirmation for the infection. If more than one infection was present, refer to the infection, which has most probably caused sepsis. If it is not possible to decide, which infection caused sepsis, than report the highest degree of confirmation.

- An infection shall be regarded as microbiologically proven, if a relevant infectious agent was proven within an appropriate timeframe (collection within 24 hours before and 24 hours after onset of infection or ICU admission) and has a causal link to the infection (allocation to a source of infection, no exogenous contamination of the sample, typical pathogen spectrum).
- Other confirmations of infection can be radiological findings with clinical symptoms, a surgical source control, abnormal urine status, or comparable findings.
- Infections are considered clinically suspected in patients when only nonspecific or indirect evidence is present, such as elevated infection levels and/or fever.

2.2 Definition of systemic inflammatory response syndrome (SIRS) according to sepsis-1 definition

2. if *1a = yes*: infection related SIRS criteria

a. Have there been at least 2 SIRS criteria due to infection present simultaneously / at the same time?	☐ 0 no<br>☐ 1 yes<br>☐ 9 unknown
b. *if a=yes*, select the according SIRS criteria present at the same time (≥ 2)	☐ tachycardia (≥90/min)<br>☐ tachypnoea (>=20/min) and/ or hypocapnia (arterial paCO2 ≤ 4,3 kPa [33 mmHg]) and / or mechanical ventilation<br>☐ leukocytosis ≥ 12000/µl or leukopenia ≤ 4000/µl and/or Normal WBC count with > 10% immature forms<br>☐ hypothermia (≤36°C) or fever (≥38°C)

Please document data of infection-related SIRS criteria only for the simultaneous occurrence of at least 2 criteria within a 24-hour time window. They should only be checked if the SIRS criteria exist due to infection.

- Tachycardia counts for a heart rate ≥90/min, which cannot be explained by other clinical causes (for exampleo tachycardia due to volume deficiency, which is regressive after volume substitution)
- Tachypnea is defined as a respiratory rate ≥20/min or an arterial paCO2 ≤4.3 kPa/33 mmHg. Ventilation includes any form of controlled or assisted ventilation. The only exceptions are the application of CPAP (continuous positive airway pressure) or NIV (noninvasive ventilation) for respiratory exercise.
- Leukocytosis/leukopenia/left shift means a leukocyte count ≥12000/µl or ≤4000/µl or more than 10% immature neutrophil granulocytes in the differential blood count.
- The core body temperature is to be used to indicate the temperature. Core temperature can be measured rectally, sublingually, via a central catheter, bladder catheter or tympanitically. When measuring an axillary temperature, 0.5°C is added to the measured value.

2.3 Presence of organ dysfunction according to sepsis-1 definition

3. *if 1a = yes*: infection-related organ dysfunction

a. After the onset of infection, did criteria referring to a new onset of infection-related organ dysfunction occur?	☐ 0 no	☐ 1 yes	☐ 9 unknown
If 3a = yes: b1. Acute encephalopathy (impaired vigilance, disorientation, restlessness, delirium)	☐ 0 no	☐ 1 yes	☐ 9 unknown
b2. thrombocytopenia (decrease in platelet count of more than 30% within 24 h or platelet count ≤ 100.000/mm^3. Acute hemorrhage or immunological causes must be ruled out)	☐ 0 no	☐ 1 yes	☐ 9 unknown
b3. arterial hypoxemia (PaO_2 ≤10 kPa (≤75 mmHg) while breathing room air or PaO_2/FiO_2-ratio ≤33 kPa (≤250 mmHg) on oxygen administation. Manifested heart- or lung disease must be ruled out as cause.)	☐ 0 no	☐ 1 yes	☐ 9 unknown
b4. renal dysfunction (diuresis of ≤ 0.5 ml/kg/h for at least 2 h despite adequate volume resuscitation and/or increase in serum creatinine level >2x the upper limit of normal)	☐ 0 no	☐ 1 yes	☐ 9 unknown
b5. metabolic acidosis (base excess ≤-5 mmol/l or lactate concentration >1.5x the upper limit of normal)	☐ 0 no	☐ 1 yes	☐ 9 unknown
b6. arterial hypotension (confirmation of infection and > 1 h systolic arterial BP ≤90 mmHg or MAP ≤70 mmHg or vasopressor administration to maintain target systolic BP of ≥90 mmHg or MAP ≥70mmHg; despite adequate volume resuscitation and not explainable by other causes)	☐ 0 no	☐ 1 yes	☐ 9 unknown

BP: blood pressure, MAP: mean arterial pressure

Please indicate any new onset of infection-related organ dysfunction or significant worsening of pre-existing organ dysfunction. All organ dysfunctions that already existed at the time of onset of infection, severe sepsis or septic shock and are attributable to another cause are not documented here (e.g. chronic kidney failure in diabetes mellitus or thrombocytopenia after trauma). Unknown is to be selected if values are unknown or have not been collected.

Acute encephalopathy

Impaired vigilance, disorientation, agitation or delirium as a result of infection must be documented. If the patient's vigilance or orientation is reduced due to other causes and/or if the patient is sedated, indicate "no" for this organ dysfunction.

Thrombocytopenia

If the platelet count is reduced due to an underlying disease, chemotherapy, or an immunological cause, or if it is a consequence of acute bleeding, this organ dysfunction is "no". If the platelet count is significantly worsened by the severe sepsis (30% reduction in platelet count), septic organ dysfunction is present.

Arterial hypoxemia

Manifest heart or lung disease must be excluded as a cause. The lowest oxygenation index (= Horovitz quotient, the worst PaO_2/FiO_2 ratio) is asked for. In case of continuous documentation of the oxygenation index (patient with ventilation), please indicate the lowest value. In case of automatic calculation or data transfer from blood gas analyzer and/or ventilator, ensure that only arterial (no venous) blood gas analyses are used and that the current FiO_2 at the time of sampling is taken into account. If no documentation of the oxygenation index is available, please always use the values of an arterial (capillary) blood gas analysis and the FiO_2 from the same point in time to calculate the oxygenation index.

Calculation of oxygenation index/Horovitz quotient:

To do this, one must determine the arterial partial pressure of oxygen (PaO_2) in the blood by means of a blood gas analysis, for example, and divide this by the inspiratory oxygen concentration, i.e. the oxygen concentration of the inhaled air (FiO_2).

$$\text{Oxygenation index (OI)} = \frac{PaO_2 \text{ in mmHg}}{FiO_2}$$

Note that the PaO_2 in kPa must first be converted to mmHg to determine the oxygenation index. Use the factor 7.5 for the conversion.

If arterial (capillary) blood gas analysis is not available, oxygen saturation SpO_2 and oxygen delivery can be used to calculate the oxygenation index. Use the following two conversion tables to determine the calculated PaO_2 and estimated FiO_2.

O_2 saturation Conversion table	
SpO_2 (%)	Calculated PaO_2 (mmHg)
80	44
81	45
82	46
83	47
84	49
85	50
86	52
87	53
88	55
89	57
90	60
91	62
92	65
93	69
94	73
95	79
96	86
97	96
98	112
99	145

For conversion using the conversion table, enter the O_2 saturation from the finger sensor and read the calculated arterial PaO_2 in mmHg in the table for this value.

Method	*O_2 flow (l/min)*	*Estimated FiO_2 (%)*
Nasal probe, nasal cannula	1<br>2<br>3<br>4<br>5<br>6	24<br>28<br>32<br>36<br>40<br>44
Nasopharyngeal catheter	4<br>5<br>6	40<br>50<br>60
Face mask	5<br>6-7<br>7-8	40<br>50<br>60
Face mask with reservoir	6<br>7<br>8<br>9<br>10	60<br>70<br>80<br>90<br>95

To determine the estimated FiO_2, the oxygen delivery is determined either via a nasal probe, nasopharyngeal catheter, or face mask in l/min and the estimated FiO_2 is read in the table.

Dividing the calculated arterial PaO_2 in mmHg by the estimated FiO_2 gives an approximation of the PaO_2/FiO_2 ratio – the oxygenation index.

Renal dysfunction

A diuresis of ≤0.5 ml/kg/h for at least 2 h despite adequate volume substitution and/or a more than twofold increase in serum creatinine above the locally usual reference range is considered as renal organ dysfunction. If creatinine is chronically elevated or the patient had a pre-existing dependence on renal replacement therapy, indicate "no" for this organ dysfunction unless there is a significant deterioration in renal function with a decrease in self-diuresis below the specified value of ≤0.5 ml/kg/h for at least 2 h despite adequate volume substitution.

Metabolic acidosis

Metabolic acidosis is defined by a base excess of ≤ -5 mmol/l or a lactate concentration, which is >1.5 times above the locally usual reference range. If acidosis is present due to other respiratory or metabolic causes, indicate "no" for this organ dysfunction.

Arterial hypotension (septic shock according to sepsis-1 definition)

If arterial hypotension exists with systolic blood pressure ≤90 mmHg or mean arterial pressure of ≤70 mmHg for at least 1 hour or if administration of vasopressors (dopamine at least 5µg/kg/min, epinephrine, norepinephrine, phenylephrine, or vasopressin in any dose) is required to maintain systolic blood pressure of at least 90 mmHg or mean arterial pressure at least 70 mmHg, indicate "yes." Note that adequate hydration was provided and other causes of shock were excluded.

2.4 Presence of organ dysfunction according to sepsis-3 definition

4. *if 1a = yes*: infection-related increase in SOFA score ≥2 points

a. infection-related SOFA increase ≥ 2 pt.	☐ 0 no<br>☐ 1 yes<br>☐ 2 evaluation impossible because no previous values available<br>☐ 3 evaluation impossible because no values were measured

b. *If 4a = yes*: list the SOFA values referring to the respective organ systems for the timepoint PRIOR TO the first infection-rated SOFA score increase of at least 2 pt.

**Organ system**	**0**	**1**	**2**	**3**	**4**	
**Respiration** PaO2/FiO2, mmHg	☐	☐<400	☐<300	☐<200 and respiratory support	☐<100 and respiratory support	☐unknown
**Central nervous system** Glasgow Coma Score (GCS)	☐	☐13–14	☐10–12	☐6–9	☐<6	☐unknown
**Cardiovascular system** (adrenergic agents administered for at least 1 h, doses in µg/kg min)	☐	☐MAP <70 mmHg	☐Dopamine ≤5 or dobutamine (any dose)	☐Dopamin >5 or epinephrin ≤0.1 or norepinephrin ≤0.1	☐Dopamin >15 or epinephrin >0.1 or norepinephrin >0.1	☐unknown
**Liver** Bilirubin mg/dl (µmol/l)	☐	☐1.2–1.9 (20–32)	☐2.0–5.9 (33–101)	☐6.0–11.9 (102–204)	☐>12.0 (>204)	☐unknown
**Coagulation** Platelets /µl	☐	☐<150.000	☐<100.000	☐<50.000	☐<20.000	☐unknown
**Renal system** Creatinine, mg/dl (µmol/l) or urine output	☐	☐1.2–1,9 (110–170)	☐2.0–3,4 (171–299)	☐3.5–4,9 (300–440) or urine output < 500 ml/d)	☐> 5.0 (> 440) (or urine output < 200 ml/d)	☐unknown

c. *If 4a = yes:* list the SOFA values referring to the respective organ systems for the time point AFTER the first infection-related SOFA-score increase of at least 2 pt.

**Organ system**	**0**	**1**	**2**	**3**	**4**	
**Respiration** PaO2/FiO2, mmHg	☐	☐<400	☐<300	☐<200 and respiratory support	☐<100 and respiratory support	☐unknown
**Central nervous system** Glasgow Coma Score (GCS)	☐	☐13–14	☐10–12	☐6–9	☐<6	☐unknown
**Cardiovascular system** (adrenergic agents administered for at least 1 h, doses in µg/kg min)	☐	☐MAP <70 mmHg	☐Dopamine ≤5 or dobutamine (any dose)	☐Dopamin >5 or epinephrin ≤0.1 or norepinephrin ≤0.1	☐Dopamin >15 or epinephrin >0.1 or norepinephrin >0.1	☐unknown
**Liver** Bilirubin mg/dl (µmol/l)	☐	☐1.2–1.9 (20–32)	☐2.0–5.9 (33–101)	☐6.0–11.9 (102–204)	☐>12.0 (>204)	☐unknown
**Coagulation** Platelets /µl	☐	☐<150.000	☐<100.000	☐<50.000	☐<20.000	☐unknown
**Renal system** Creatinine, mg/dl (µmol/l) or urine output	☐	☐1.2–1,9 (110–170)	☐2.0–3,4 (171–299)	☐3.5–4,9 (300–440) or urine output < 500 ml/d)	☐> 5.0 (> 440) (or urine output < 200 ml/d)	☐unknown

Check whether there was an infection-related increase in SOFA score of at least 2 points at any time during the stay after the onset of infection. Document the SOFA score for the time BEFORE the onset of the first infection-related SOFA increase by ≥2 points and also AFTER the first infection-related SOFA increase ≥ 2 points. For nervous system assessment, please use the Glasgow Coma Scale.

2.5 Presence of septic shock according to sepsis-3 definition

5. *if 1a = yes*: infection-related criteria for septic shock (sepsis-3)

a. After onset of infection: Did septic shock criteria according to sepsis-3 were present simultaneously? (increase in serum lactate to > 2mmol/l; persistent hypotension demanding vasopressor administration to maintain MAP ≥65 mmHg)	☐ 1 yes, both criteria were present simultaneously<br>☐ 2 persistent hypotension, but no increase in serum lactate<br>☐ 3 persistent hypotension, but no information regarding serum lactate available<br>☐ 4 increase in serum lactate, but no persistent hypotension<br>☐ 5 increase in serum lactate, but no information regarding blood pressure<br>☐ 6 none of the criteria present (or one not present and the other unknown)<br>☐ 7 both criteria unknown

The criteria must have been present simultaneously (in a 24 h interval). For the increase in lactate, only an infection-related increase to >2mmol/l should be reported. When assessing hypotension with vasopressor therapy, it is important to note that adequate hydration occurred and other causes of shock were excluded. If information evaluating one or both criteria is not available in the record, indicate as "not measured/unknown." If one of the two values was normal (no hypotension or no increase in serum lactate) and the other value is missing, then the category "None of the criteria was present" should be checked (for explanation: if one criterion was certainly not present, then the criterion for shock is not fulfilled in any case - whether the information on the other criterion is missing does not matter).

3 Definition of variables in administrative health data

3.1 Explanation

Analyses were based on data in the format according to §21-KHEntgG, which defines the format of data used for billing of hospitals in the German DRG-system. These data provide information in different data tables in csv-files. The following data tables were used: a) “FALL”: general information on the case, b) “ICD”: information on codes according to the German Modification of the ICD-10, c) “OPS”: information on the codes for surgeries and procedures in Germany (“Operationen- und Prozedurenschlüssel”). The following sections describe the definition of variables, which ware based on these different data tables, as well as variables, which were calculated using previously defined variables.

3.2 Variable derived by recoding of variables in data table „FALL“

The following variables were based on recoding information in data table “FALL”. The presented definitions are using the syntax of the statistical software R.

Label	Hospital mortality
Variable	kh_mort_neu
Definition	kh_mort_neu <- as.numeric(Entlassungsgrund == “079” | Entlassungsgrund == “79”)

Label	Reason for admission
Variable	aufn_anl_ges_neu
Definition	aufn_anl_ges_neu <- rep(NA,nrow(fall)); aufn_anl_ges_neu[Aufnahmeanlass== “E” | Aufnahmeanlass== “Z”] <- 1; aufn_anl_ges_neu[Aufnahmeanlass== “N”] <- 2; aufn_anl_ges_neu[Aufnahmeanlass== “V”] <- 3; aufn_anl_ges_neu[Aufnahmeanlass== “A”] <- 4; aufn_anl_ges_neu[Aufnahmeanlass== “R”] <- 5; aufn_anl_ges_neu <- factor(aufn_anl_ges_neu,levels=1:5,labels=c(“Referral by physician”, “Emergency admission”,“Transfer from other hospitals (> 24h treatment)”, “Transfer from other hospital (< 24h treatment)”, “Transfer from rehabilitation”));aufn_anl_ges_neu <- factor(aufn_anl_ges_neu)

Label	Sex: female
Variable	sex_num_neu
Definition	sex_num_neu <- as.numeric(Geschlecht==“w”); sex_num_neu[Geschlecht==“u”] <- NA

3.3 Variables based on ICD-codes

The following variables were defined based on the ICD-codes in data table “ICD“. Each variable was defined as an indicator variable (0: condition not present, 1: condition present), if at least one of the listed ICD-codes was present in primary or secondary diagnosis. If ICD-codes are listed with less than three or four characters, all subordinate ICD-codes are included. CCI: Charlson comorbidity index. ECI: Elixhauser comorbidity index.

Label	Asplenia, acquired
Variable	asplenie_erw_neu
ICD-Codes	Q890

Label	Asplenia, congenital
Variable	asplenie_ang_neu
ICD-Codes	D730

Label	CCI: AIDS/HIV
Variable	cha_hiv_neu
ICD-Codes	B20; B21; B22; B24

Label	CCI: Any malignancy
Variable	cha_mal_neu
ICD-Codes	C00; C01; C02; C03; C04; C05; C06; C07; C08; C09; C10; C11; C12; C13; C14; C15; C16; C17; C18; C19; C20; C21; C22; C23; C24; C25; C26; C30; C31; C32; C33; C34; C37; C38; C39; C40; C41; C43; C45; C46; C47; C48; C49; C50; C51; C52; C53; C54; C55; C56; C57; C58; C60; C61; C62; C63; C64; C65; C66; C67; C68; C69; C70; C71; C72; C73; C74; C75; C76; C81; C82; C83; C84; C85; C86; C88; C90; C91; C92; C93; C94; C95; C96; C97

Label	CCI: Cerebrovascular disease
Variable	cha_cvd_neu
ICD-Codes	G45; G46; H340; I60; I61; I62; I63; I64; I65; I66; I67; I68; I69

Label	CCI: Chronic pulmonary disease
Variable	cha_cpd_neu
ICD-Codes	I278; I279; J40; J41; J42; J43; J44; J45; J46; J47; J60; J61; J62; J63; J64; J65; J66; J67; J684; J701; J703

Label	CCI: Congestive heart failure
Variable	cha_chf_neu
ICD-Codes	I099; I110; I130; I132; I255; I420; I425; I426; I427; I428; I429; I43; I50; P290

Label	CCI: Dementia

Variable cha_dem_neu
ICD-Codes F00; F01; F02; F03; F051; G30; G311

Label CCI: Diabetes with chronic complication
Variable cha_dwc_neu
ICD-Codes E102; E103; E104; E105; E107; E112; E113; E114; E115; E117; E122; E123; E124; E125; E127; E132; E133; E134; E135; E137; E142; E143; E144; E145; E147

Label CCI: Diabetes without chronic complication
Variable cha_dwoc_neu
ICD-Codes E100; E101; E106; E108; E109; E110; E111; E116; E118; E119; E120; E121; E126; E128; E129; E130; E131; E136; E138; E139; E140; E141; E146; E148; E149

Label CCI: Hemiplegia or paraplegia
Variable cha_hemi_neu
ICD-Codes G041; G114; G801; G802; G81; G82; G830; G831; G832; G833; G834; G835; G839

Label CCI: Metastatic solid tumor
Variable cha_mts_neu
ICD-Codes C77; C78; C79; C80

Label CCI: Mild liver disease
Variable cha_mild_neu
ICD-Codes B18; K700; K701; K702; K703; K709; K713; K714; K715; K717; K73; K74; K760; K762; K763; K764; K768; K769; Z944

Label CCI: Moderate or severe liver disease
Variable cha_msld_neu
ICD-Codes I85; I864; I982; I983; K704; K711; K721; K729; K765; K766; K767

Label CCI: Myocardial infarction
Variable cha_mi_neu
ICD-Codes I21; I22; I252

Label CCI: Peptic ulcer disease
Variable cha_pud_neu
ICD-Codes K25; K26; K27; K28

Label CCI: Peripheral vascular disease
Variable cha_pvd_neu
ICD-Codes I70; I71; I731; I738; I739; I771; I790; I792; K551; K558; K559; Z958; Z959

Label CCI: Renal disease
Variable cha_red_neu
ICD-Codes I120; I131; N032; N033; N034; N035; N036; N037; N052; N053; N054; N055; N056; N057; N18; N19; N250; Z49; Z940; Z992

Label CCI: Rheumatic disease
Variable cha_rhd_neu
ICD-Codes M05; M06; M315; M32; M33; M34; M351; M353; M360

Label ECI: AIDS/HIV
Variable elix_hiv_neu
ICD-Codes B20; B21; B22; B24

Label ECI: Alcohol abuse
Variable elix_alc_neu
ICD-Codes E52; F10; G621; I426; K292; K700; K703; K709; T51; Z502

Label ECI: Blood loss anemia
Variable elix_blan_neu
ICD-Codes D500

Label ECI: Cardiac arrhythmias
Variable elix_car_neu
ICD-Codes I441; I442; I443; I456; I459; I47; I48; I49; R000; R001; R008; T821; Z450; Z950

Label ECI: Chronic pulmonary disease
Variable elix_cpd_neu
ICD-Codes I278; I279; J40; J41; J42; J43; J44; J45; J46; J47; J60; J61; J62; J63; J64; J65; J66; J67; J684; J701; J703

Label ECI: Coagulopathy
Variable elix_coag_neu
ICD-Codes D65; D66; D67; D68; D691; D693; D694; D695; D696

Label ECI: Congestive heart failure
Variable elix_chf_neu

ICD-Codes	I099; I255; I420; I425; I426; I427; I428; I429; I43; I50; P290

Label	ECI: Deficiency anemia
Variable	elix_defan_neu
ICD-Codes	D508; D509; D51; D52; D53

Label	ECI: Depression
Variable	elix_dep_neu
ICD-Codes	F204; F313; F314; F315; F32; F33; F341; F412; F432

Label	ECI: Diabetes, complicated
Variable	elix_dwc_neu
ICD-Codes	E102; E103; E104; E105; E106; E107; E108; E112; E113; E114; E115; E116; E117; E118; E122; E123; E124; E125; E126; E127; E128; E132; E133; E134; E135; E136; E137; E138; E142; E143; E144; E145; E146; E147; E148

Label	ECI: Diabetes, uncomplicated
Variable	elix_dwoc_neu
ICD-Codes	E100; E101; E109; E110; E111; E119; E120; E121; E129; E130; E131; E139; E140; E141; E149

Label	ECI: Drug abuse
Variable	elix_dra_neu
ICD-Codes	F11; F12; F13; F14; F15; F16; F18; F19

Label	ECI: Fluid and electrolyte disorders
Variable	elix_fed_neu
ICD-Codes	E222; E86; E87

Label	ECI: Hypertension, complicated
Variable	elix_htc_neu
ICD-Codes	I11; I12; I13; I15

Label	ECI: Hypertension, uncomplicated
Variable	elix_htu_neu
ICD-Codes	I10

Label	ECI: Hypothyroidism
Variable	elix_hth_neu
ICD-Codes	E00; E01; E02; E03; E890

Label	ECI: Liver disease
Variable	elix_ld_neu
ICD-Codes	B18; I85; I864; I982; I983; K70; K711; K713; K714; K715; K717; K72; K73; K74; K760; K762; K763; K764; K765; K766; K767; K768; K769

Label	ECI: Lymphoma
Variable	elix_lymph_neu
ICD-Codes	C81; C82; C83; C84; C85; C86; C88; C900; C902; C903; C96

Label	ECI: Metastatic cancer
Variable	elix_mts_neu
ICD-Codes	C77; C78; C79; C80

Label	ECI: Obesity
Variable	elix_obes_neu
ICD-Codes	E66

Label	ECI: Other neurological disorders
Variable	elix_neur_neu
ICD-Codes	G10; G11; G12; G13; G20; G21; G22; G254; G255; G312; G318; G319; G32; G35; G36; G37; G40; G41; G931; G934; R470; R56

Label	ECI: Paralysis
Variable	elix_par_neu
ICD-Codes	G041; G114; G801; G802; G81; G82; G830; G831; G832; G833; G834; G835; G839

Label	ECI: Peptic ulcer disease excluding bleeding
Variable	elix_pud_neu
ICD-Codes	K257; K259; K267; K269; K277; K279; K287; K289

Label	ECI: Peripheral vascular disorders
Variable	elix_pvd_neu
ICD-Codes	I70; I71; I731; I738; I739; I771; I790; I792; K551; K558; K559; Z958; Z959

Label	ECI: Psychoses
Variable	elix_psy_neu
ICD-Codes	F20; F22; F23; F24; F25; F28; F29; F302; F312; F315

Label	ECI: Pulmonary circulation disorders
Variable	elix_pcd_neu
ICD-Codes	I26; I27; I280; I288; I289

Label	ECI: Renal failure
Variable	elix_rf_neu
ICD-Codes	N18; N19; N250; Z49; Z940; Z992

Label	ECI: Rheumatoid arthritis/collagen vascular diseases
Variable	elix_rhd_neu
ICD-Codes	L940; L941; L943; M05; M06; M08; M120; M123; M30; M310; M311; M312; M313; M32; M33; M34; M35; M45; M461; M468; M469

Label	ECI: Solid tumor without metastasis
Variable	elix_mal_neu
ICD-Codes	C00; C01; C02; C03; C04; C05; C06; C07; C08; C09; C10; C11; C12; C13; C14; C15; C16; C17; C18; C19; C20; C21; C22; C23; C24; C25; C26; C30; C31; C32; C33; C34; C37; C38; C39; C40; C41; C43; C45; C46; C47; C48; C49; C50; C51; C52; C53; C54; C55; C56; C57; C58; C60; C61; C62; C63; C64; C65; C66; C67; C68; C69; C70; C71; C72; C73; C74; C75; C76; C97

Label	ECI: Valvular disease
Variable	elix_vd_neu
ICD-Codes	A520; I05; I06; I07; I08; I091; I098; I34; I35; I36; I37; I38; I39; Q230; Q231; Q232; Q233; Z952; Z953; Z954

Label	ECI: Weight loss
Variable	elix_wl_neu
ICD-Codes	E40; E41; E42; E43; E44; E45; E46; R634; R64

Label	Explicit sepsis-1
Variable	sepsis_neu_ohneO
ICD-Codes	A021; A200; A207; A217; A227; A241; A267; A282; A327; A391; A392; A393; A394; A40; A41; A427; A483; A499; A548; B007; B376; B377; B49; P36; R572; R650; R651

Label	Explicit septic shock-1 (also used for septic shock-3)
Variable	explsepschock_neu
ICD-Codes	R572

Label	Infection
Variable	infcode_neu
ICD-Codes	A00; A01; A02; A03; A04; A05; A06; A07; A08; A09; A15; A16; A17; A18; A19; A20; A21; A22; A23; A24; A25; A26; A27; A28; A32; A36; A37; A38; A39; A40; A41; A42; A43; A44; A46; A48; A49; A50; A54; A55; A56; A59; A65; A690; A691; A692; A698; A699; A74; A75; A77; A78; A79; A80; A81; A83; A84; A85; A86; A87; A88; A89; A90; A91; A92; A93; A94; A95; A96; A97; A98; A99; B00; B01; B02; B03; B04; B05; B06; B07; B08; B09; B25; B26; B27; B33; B34; B37; B38; B39; B40; B41; B42; B43; B44; B45; B46; B47; B48; B49; B50; B51; B52; B53; B54; B55; B58; B60; B64; B67; B95; B96; B97; B98; B99; G00; G01; G02; G03; G04; G05; G06; G07; G08; H050; H602; H700; I32; I33; I38; I39; I40; I41; I80; I981; J01; J02; J03; J04; J05; J06; J09; J10; J11; J12; J13; J14; J15; J16; J17; J18; J20; J21; J22; J36; J390; J391; J440; J441; J85; J86; K35; K36; K37; K5702; K5703; K5712; K5713; K5722; K5723; K5732; K5733; K5742; K5743; K5752; K5753; K5782; K5783; K5792; K5793; K61; K630; K631; K65; K67; K750; K751; K770; K810; L02; L03; L04; L05; L08; M00; M01; M86; N10; N151; N159; N30; N34; N390; N41; N45; N482; N49; N61; N70; N71; N72; N73; N74; N75; N76; N77; N980; O030; O035; O040; O045; O050; O055; O060; O065; O070; O075; O080; O23; O411; O753; O85; O86; O883; O91; O98; P23; P240; P248; P249; P35; P36; P37; P38; P39; P77; P781; R572; R650; R651; T802; T814; T826; T827; T835; T836; T845; T846; T847; T857; T880; U6900; U6940

Label	Leukaemia
Variable	elix_new_leuk_neu
ICD-Codes	C901; C91; C92; C93; C94; C95

Label	Organ dysfunction
Variable	odfcode_neu
ICD-Codes	D65; D688; D689; D695; D696; E872; F05; G931; G934; I959; J80; J960; J969; J984; K720; K727; K762; K763; N17; N19; R060; R068; R40; R572; R578; R579; R651

Label	Systemic inflammatory response syndrome with organ dysfunction
Variable	explseveresepsis_neu
ICD-Codes	R651

Label	Transplanted organ status
Variable	organtr_prev_neu
ICD-Codes	Z94

3.4 Variables based on OPS-codes

The following variables were defined based on the OPS-codes in data table "OPS". Each variable was defined as an indicator variable (0: condition not present, 1: condition present), if at least one of the listed OPS-codes was

present. If OPS-codes do not present the full number of possible characters, all subordinate OPS-codes are included.

Label	Chemotherapy
Variable	ctx_neu
OPS-Codes	854

Label	Complex intensive care treatment
Variable	ops_its_neu
OPS-Codes	8980; 898c; 898d; 898f

Label	Stroke treatment
Variable	stroke_neu
OPS-Codes	8981; 898b

3.5 Variables calculated using previously defined variables.

The following variables were calculated using previously defined variables given above. Definitions use the syntax of the statistical software R.

Label	Asplenia
Variable	asplenie_neu
Definition	asplenie_neu <- as.numeric(asplenie_erw_neu==1 | asplenie_ang_neu==1)

Label	Charlson comorbidity index
Variable	chacmi_neu
Definition	chacmi_neu <- (cha_mi_neu*1)+ (cha_chf_neu*1)+ (cha_pvd_neu*1)+ (cha_cvd_neu*1)+ (cha_dem_neu*1)+ (cha_cpd_neu*1)+ (cha_rhd_neu*1)+ (cha_pud_neu*1)+ (cha_mild_neu*1)+ (cha_dwoc_neu*1)+ (cha_dwc_neu*2)+ (cha_hemi_neu*2)+ (cha_red_neu*2)+ (cha_mal_neu*2)+ (cha_msld_neu*3)+ (cha_mts_neu*6)+ (cha_hiv_neu*6)

Label	Elixhauser comorbidity index
Variable	elixcmi_neu
Definition	elixcmi_neu <- (elix_chf_neu*7) + (elix_car_neu*5) + (elix_vd_neu*-1) + (elix_pcd_neu*4) + (elix_pvd_neu*2) + (elix_htu_neu*0) + (elix_htc_neu*0) + (elix_par_neu*7) + (elix_neur_neu*6) + (elix_cpd_neu*3) + (elix_dwoc_neu*0) + (elix_dwc_neu*0) + (elix_hth_neu*0) + (elix_rf_neu*5) + (elix_ld_neu*11) + (elix_pud_neu*0) + (elix_hiv_neu*0) + (elix_lymph_neu*9) + (elix_mts_neu*12) + (elix_mal_neu*4) + (elix_rhd_neu*0) + (elix_coag_neu*3) + (elix_obes_neu*-4) + (elix_wl_neu*6) + (elix_fed_neu*5) + (elix_blan_neu*-2) + (elix_defan_neu*-2) + (elix_alc_neu*0)+ (elix_dra_neu*-7) + (elix_psy_neu*0) + (elix_dep_neu*-3)

Label	Explicit severe sepsis-1 (also used for sepsis-3)
Variable	explseveresepsisshock_neu
Definition	explseveresepsisshock_neu <- as.numeric(explseveresepsis_neu==1 | explsepschock_neu==1)

Label	Implicit severe sepsis-1 (Angus definition)
Variable	inf_odf_neu
Definition	inf_odf_neu <- as.numeric(infcode_neu==1 & odfcode_neu==1)

Label	Implicit severe sepsis-1 (modified Martin definition)
Variable	sepsis_odf_neu
Definition	sepsis_odf_neu <- as.numeric(sepsis_neu_ohneO==1 & odfcode_neu==1)

4 Analysis of the German DRG-statistics

4.1 Background

German national IAHD have been used previously to calculate yearly incidence proportions of sepsis [1, 3]. For the year 2015, Fleischmann et al. identified 136,542 cases with ICD-codes for severe sepsis-1 among all hospitalizations represented in the national DRG-statistics, corresponding to an incidence of 158 per 100,000 inhabitants [3].

4.2 Design

A retrospective observational study was conducted based on national IAHD to assess sepsis incidence in Germany for the year 2017.

4.3 Setting

In Germany, hospitals are reimbursed based on a diagnosis related groups (DRG) system. Every year a standardized data set is transferred to the federal Institute of Hospital Reimbursement (*Institut für das Entgeltsystem im Krankenhaus*; InEK) by every hospital providing acute care (legal base: §21 KHEntgG). These data are passed to the Federal Bureau of Statistics and can be analysed for research purposes [12].

4.4 Sample

The same inclusion criteria as described for the validation study (inpatient cases, DRG-billing, age of at least 15 years) are applied to German national IAHD of the year 2017.

4.5 Procedure

National IAHD are hosted by the Federal Bureau of statistics and can be accessed via a form of remote data processing. Based on completely anonymized sample data files, statistical syntaxes are written and sent to the Federal Bureau where they are applied to the original data files. Output files are then transferred back to the researcher.

4.6 Statistical analysis

Sepsis incidencewas calculated by obtaining the number of hospital episodes with ICD-10 coded severe sepsis-1 (ICD-10-GM codes R65.1 or R57.2) and dividing them by the size of the German population with age ≥ 15 years within the same year. The size of the population was obtained from the GENESIS data base, which is also provided by the Federal Bureau of Statistics.

4.7 Results

Overall, 17,088,417 inpatient, DRG-billed hospital episodes of patients ≥ 15 years of age were documented in the national DRG-statistics for 2017. Explicit ICD-10-GM codes for severe sepsis-1 were present in 148,288 (0.87%) of these cases. Based on the GENESIS data-base, Germany had 71.6 million inhabitants aged 15 years or older in 2017. This led to an estimate for the incidence of severe sepsis-1 of 207/100,000 inhabitants in this age spectrum. The hospital mortality of coded severe sepsis-1 was 40.3% (N = 59,792 deaths).

5 Supplemental Figures

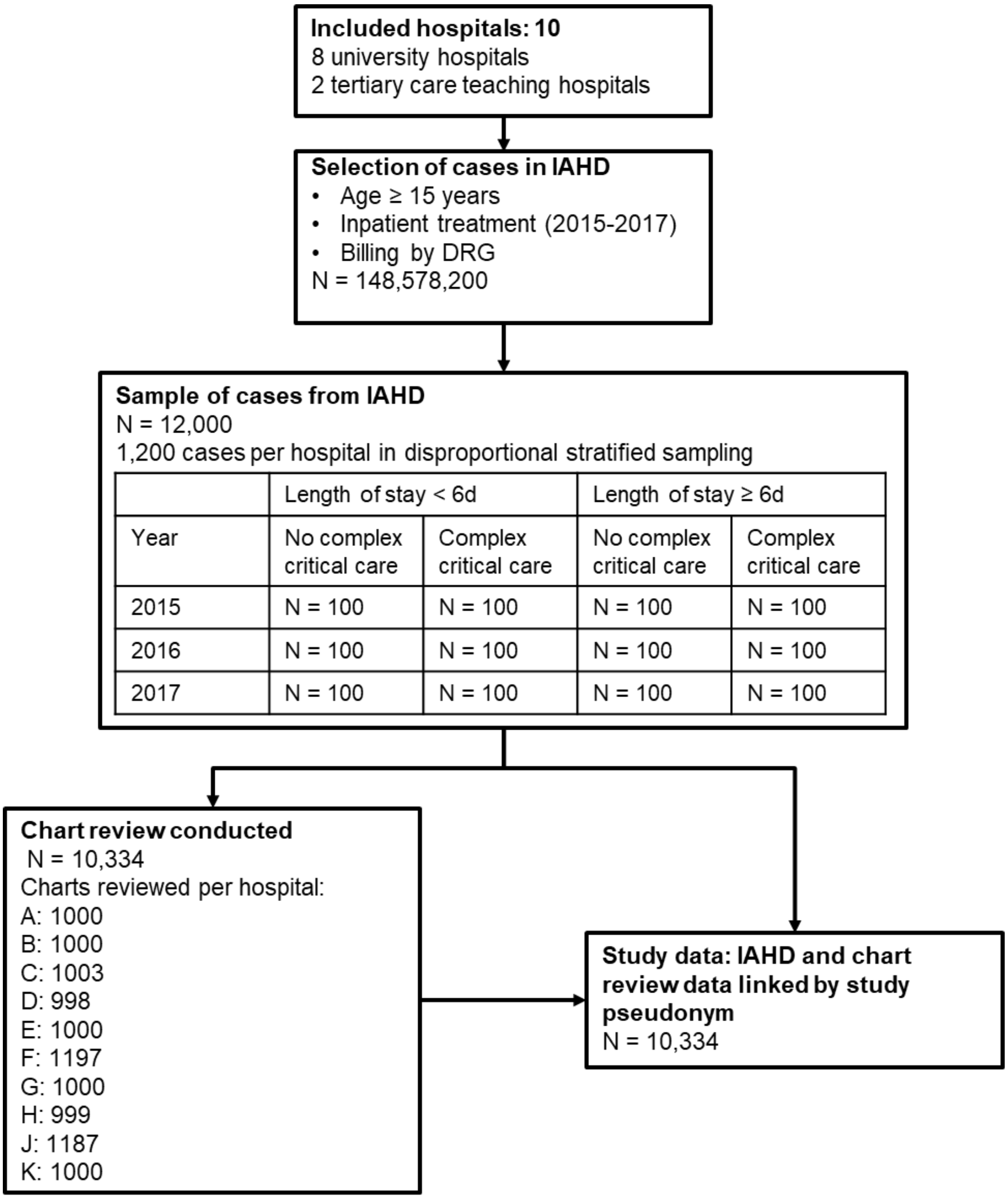

SFig. 1 Study flow chart.
IAHD: inpatient administrative health data. Explanation on conduction of chart review: N = 1,000 cases should be documented per hospital; N = 1,200 cases were sampled in case of unavailable charts; to assure representativeness and avoid bias by learning effects, the review of charts was conducted in random order.

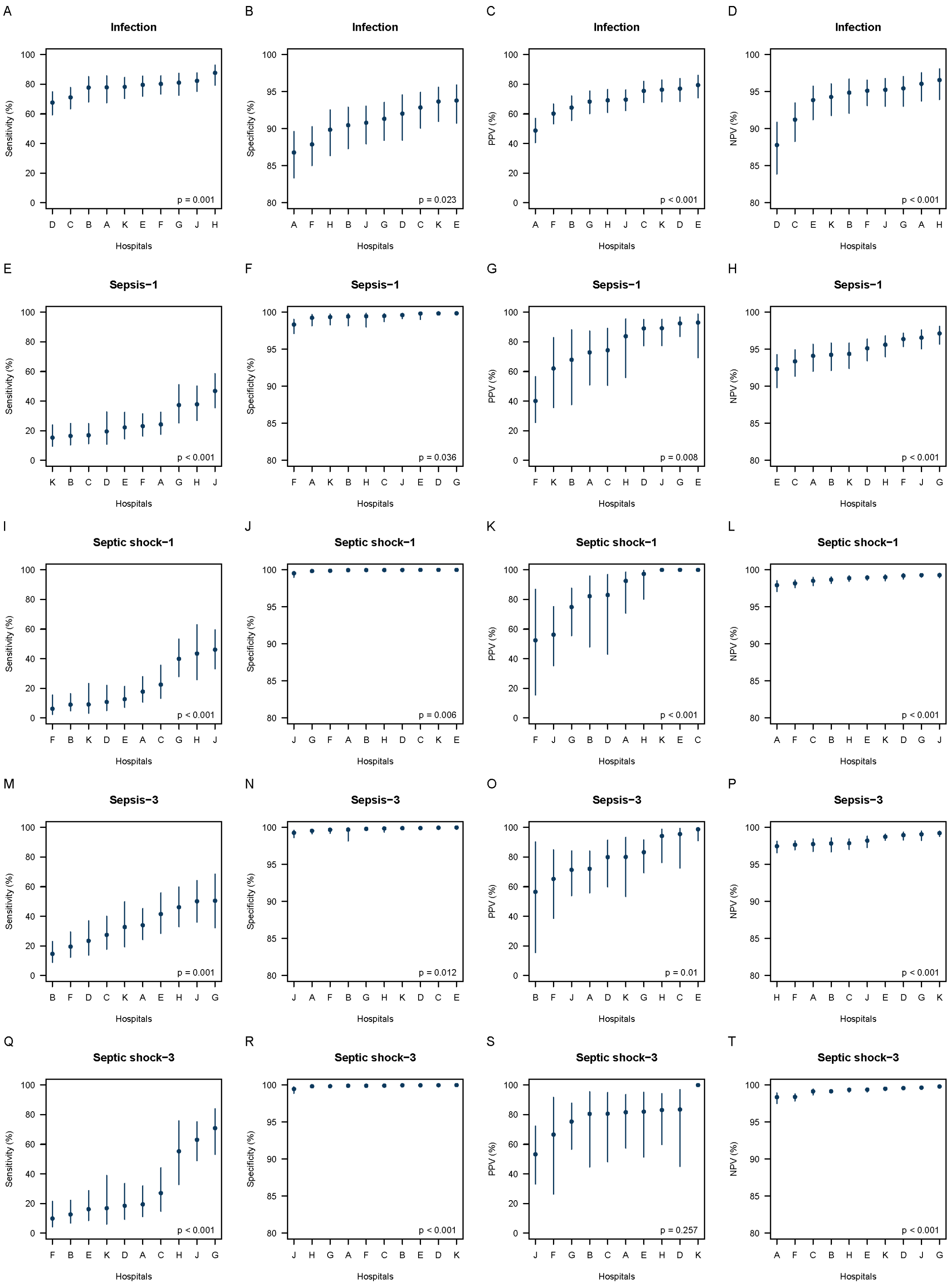

SFig. 2 Predictive accuracy of explicit codes for infection and sepsis in inpatient administrative health data.

Estimates adjusted for sampling weights and clustering. P-values obtained by Rao-Scott Pearson $\chi 2$-Test with satterthwaite approximation.

6 Supplemental Tables

STable 1 Descriptive statistics of definition criteria of sepsis.

Definition criteria	Categories	Sample: N (%)	Weighted %
Presence of ≥ 2 SIRS criteria	no infection	6880 (66.3)	79.4
	unknown	185 (1.8)	2
	no	1467 (14.1)	12.3
	yes	1852 (17.9)	6.4
Presence of organ dysfunction	no infection	6880 (66.3)	79.4
	unknown	198 (1.9)	2
	no	1875 (18.1)	14.7
	yes	1431 (13.8)	3.9
Infection related hypotension	no infection	6880 (66.3)	79.4
	unknown	232 (2.2)	2.2
	no	2358 (22.7)	16.7
	yes	914 (8.8)	1.7
Any sepsis-1 criterion unknown	yes	305 (2.9)	3
SOFA increase ≥ 2	no infection	6880 (66.3)	79.4
	unknown previous SOFA-values	318 (3.1)	1.4
	unknown SOFA-values	263 (2.5)	3.5
	no	1848 (17.8)	13.3
	yes	1075 (10.4)	2.4
Septic-shock-3 criteria	no infection	6880 (66.3)	79.4
	hypotension yes, lactate unknown	14 (0.1)	0
	lactate yes, bloodpressure unknown	8 (0.1)	0
	both criteria unknown	416 (4)	4.8
	hypotension yes, lactate no	263 (2.5)	0.5
	lactate yes, hypotenion no	216 (2.1)	0.7
	both no	1933 (18.6)	13.5
	both yes	653 (6.3)	1.1
Any sepsis-3 criterion unknown	yes	764 (7.4)	6.5

Weighted % are adjusted for sampling weights and clustering.

STable 2 Accuracy of identification of severe sepsis-1 stratified by year.

	Year		
	**2015**	**2016**	**2017**
Sensitivity	37.9% [17.5%, 63.7%]	35.9% [22.1%, 52.4%]	29.4% [20.3%, 40.6%]
Specificity	99.8% [99.5%, 99.9%]	99.7% [99.4%, 99.9%]	99.8% [99.6%, 99.9%]
PPV	84.1% [52.2%, 96.1%]	80.6% [75.4%, 84.9%]	85.7% [72.1%, 93.3%]
NPV	98% [97.6%, 98.3%]	97.8% [96.7%, 98.5%]	97.7% [97%, 98.2%]

Estimates are presented as relative frequencies (%) along with their 95% confidence intervals and were calculated with adjustment for sampling weights and clustering.

STable 3 Accuracy of identification of cases with severe sepsis-1 by indirect coding abstraction strategies.

	**Severe sepsis-1 according to chart review**	**Modified Martin definition (ICD-10 codes for sepsis & ICD-10 codes for organ dysfunction)**	**Angus definition (ICD-10 codes for infection & ICD-10 codes for organ dysfunction**
Sensitivity	-	40.5% [30.3%, 51.5%]	72.7% [63.8%, 80.1%]
Specificity	-	99.5% [99.3%, 99.7%]	95.4% [94.8%, 96.0%]
PPV	-	74.0% [61.2%, 83.7%]	35.0% [28.0%, 42.7%]
NPV	-	98.0% [97.6%, 98.4%]	99.0% [98.8%, 99.2%]
Proportion of hospital admissions	3.3% [2.6%, 4.1%]	1.8% [1.3%, 2.5%]	6.8% [5.8%, 7.9%]
Hospital mortality	27.8% [21.0%, 35.8%]	34.0% [25.8%, 43.3%]	17.4% [12.6%, 23.4%]

Estimates are presented as relative frequencies (%) along with their 95% confidence intervals and were calculated with adjustment for sampling weights and clustering.

STable 4 Accuracy of identification of risk factors for sepsis-related mortality in inpatient administrative health data

Risk factor	N missings	Proportion (coding in IAHD)	Proportion (reference standard)	Sensitivity	Speci-ficity	PPV	NPV
CCI: Myocardial infarction	16/1310	11.2% [7.6%, 16.0%]	14.6% [10.2%, 20.5%]	56.1% [43.2%, 68.3%]	96.6% [95.2%, 97.6%]	73.6% [66.5%, 79.7%]	92.8% [88.7%, 95.4%]
ECI: Congestive heart failure	26/1310	28.3% [23.5%, 33.7%]	23.8% [14.8%, 35.9%]	67.4% [48.8%, 81.8%]	83.9% [79.0%, 87.9%]	56.7% [46.0%, 66.8%]	89.2% [73.9%, 96.0%]
ECI: Cardiac arrhythmias	10/1310	39.2% [35.5%, 43.1%]	32.4% [22.4%, 44.2%]	82.0% [70.8%, 89.6%]	81.3% [73.8%, 87.0%]	67.7% [49.5%, 81.8%]	90.4% [80.1%, 95.7%]
ECI: Valvular disease	19/1310	15.2% [10.5%, 21.5%]	16.3% [12.3%, 21.2%]	64.5% [48.7%, 77.7%]	94.4% [91.0%, 96.5%]	69.0% [54.7%, 80.4%]	93.2% [90.5%, 95.1%]
ECI: Pulmonary circulation disorders	11/1310	7.6% [5.6%, 10.2%]	8.7% [5.6%, 13.3%]	34.3% [22.4%, 48.5%]	95.0% [92.2%, 96.8%]	39.3% [25.0%, 55.8%]	93.8% [89.3%, 96.5%]
ECI: Peripheral vascular disorders	14/1310	15.4% [12.4%, 19.0%]	14.8% [9.9%, 21.6%]	53.5% [27.2%, 77.9%]	91.3% [87.7%, 93.9%]	51.6% [37.9%, 65.1%]	91.8% [80.6%, 96.8%]
CCI: Cerebrovascular disease	11/1310	12.9% [9.8%, 16.9%]	9.7% [6.9%, 13.4%]	36.4% [23.6%, 51.4%]	89.6% [85.4%, 92.7%]	27.2% [13.3%, 47.7%]	92.9% [91.7%, 94.0%]
ECI: Hypertension, uncomplicated	17/1310	43.0% [36.6%, 49.7%]	50.0% [42.6%, 57.4%]	60.1% [51.3%, 68.3%]	74.1% [65.9%, 80.8%]	69.9% [61.2%, 77.3%]	65.0% [55.3%, 73.6%]
ECI: Hypertension, complicated	15/1310	10.5% [6.2%, 17.3%]	13.4% [8.5%, 20.4%]	24.1% [12.7%, 40.9%]	91.6% [85.9%, 95.1%]	30.7% [20.5%, 43.2%]	88.7% [81.5%, 93.3%]
CCI: Dementia	18/1310	8.8% [4.0%, 18.0%]	8.5% [3.3%, 19.9%]	78.0% [60.2%, 89.3%]	97.6% [96.2%, 98.6%]	75.3% [50.4%, 90.2%]	98.0% [94.8%, 99.2%]
ECI: Chronic pulmonary disease	18/1310	21.9% [15.8%, 29.6%]	23.9% [19.2%, 29.3%]	73.2% [55.8%, 85.5%]	94.1% [91.3%, 96.1%]	79.7% [72.6%, 85.2%]	91.8% [87.0%, 94.9%]
ECI: Rheumatoid arthritis/collagen vascular Diseases	10/1310	2.6% [1.5%, 4.6%]	3.2% [1.7%, 5.6%]	36.5% [16.8%, 62.1%]	98.5% [96.0%, 99.5%]	44.5% [15.8%, 77.4%]	97.9% [95.5%, 99.1%]
ECI: Peptic ulcer disease excluding bleeding	10/1310	0.3% [0.1%, 1.0%]	12.0% [4.6%, 28.0%]	0.9% [0.1%, 7.2%]	99.8% [99.1%, 100.0%]	38.2% [2.0%, 95.0%]	88.0% [72.0%, 95.5%]
CCI: Mild liver disease	10/1310	11.2% [9.3%, 13.4%]	6.9% [4.2%, 11.0%]	53.7% [23.6%, 81.3%]	92.0% [87.6%, 94.8%]	33.0% [12.7%, 62.5%]	96.4% [92.1%, 98.4%]
ECI: Diabetes, uncomplicated	6/1310	23.8% [18.8%, 29.6%]	19.4% [15.8%, 23.6%]	77.1% [69.5%, 83.2%]	89.1% [79.7%, 94.4%]	63.0% [42.9%, 79.4%]	94.1% [92.2%, 95.6%]
CCI: Hemiplegia or paraplegia	16/1310	10.1% [8.8%, 11.6%]	7.2% [4.9%, 10.4%]	51.5% [35.2%, 67.5%]	93.1% [90.9%, 94.8%]	36.6% [23.7%, 51.9%]	96.1% [93.0%, 97.9%]
ECI: Other neurological disorders	7/1310	16.2% [13.0%, 20.1%]	13.3% [9.0%, 19.1%]	52.1% [36.1%, 67.8%]	89.3% [86.9%, 91.3%]	42.6% [25.5%, 61.6%]	92.4% [89.5%, 94.6%]
ECI: Renal failure	8/1310	24.2% [19.2%, 30.0%]	11.1% [8.6%, 14.3%]	79.7% [56.9%, 92.1%]	82.7% [78.2%, 86.5%]	36.6% [31.1%, 42.5%]	97.0% [92.5%, 98.8%]
ECI: Diabetes, complicated	7/1310	7.5% [5.7%, 9.9%]	6.5% [3.9%, 10.5%]	58.9% [42.5%, 73.5%]	96.0% [93.4%, 97.7%]	50.8% [27.9%, 73.4%]	97.1% [94.7%, 98.4%]
ECI: Hypothyroidism	21/1310	9.2% [7.3%, 11.5%]	10.7% [7.6%, 14.7%]	35.9% [26.0%, 47.2%]	94.0% [91.4%, 95.8%]	41.5% [30.2%, 53.8%]	92.5% [88.4%, 95.2%]
ECI: Solid tumor without metastasis	14/1310	16.4% [12.6%, 21.1%]	14.0% [10.8%, 17.9%]	60.8% [44.7%, 74.9%]	90.8% [86.9%, 93.6%]	51.8% [37.7%, 65.6%]	93.5% [90.3%, 95.6%]
Leukaemia	6/1310	2.7% [1.5%, 4.6%]	2.9% [1.8%, 4.8%]	81.8% [46.5%, 95.9%]	99.7% [99.0%, 99.9%]	90.0% [55.9%, 98.4%]	99.5% [98.4%, 99.8%]
ECI: Lymphoma	5/1310	5.2% [1.9%, 13.6%]	5.8% [2.2%, 14.8%]	85.0% [65.2%, 94.5%]	99.8% [99.1%, 99.9%]	95.6% [68.9%, 99.5%]	99.1% [98.1%, 99.6%]

CCI: Moderate or severe liver disease	5/1310	3.3% [1.9%, 5.7%]	3.0% [1.9%, 4.8%]	52.9% [22.8%, 81.1%]	98.2% [95.6%, 99.3%]	48.2% [18.9%, 78.7%]	98.5% [96.7%, 99.4%]
ECI: Metastatic cancer	16/1310	9.0% [6.1%, 13.1%]	7.6% [4.9%, 11.7%]	83.3% [76.1%, 88.6%]	97.1% [95.7%, 98.1%]	70.6% [59.9%, 79.3%]	98.6% [97.6%, 99.2%]
ECI: AIDS/HIV	126/1310	0.1% [0.0%, 0.7%]	0.4% [0.2%, 0.8%]	21.6% [0.8%, 90.8%]	100.0% [100.0%, 100.0%]	100.0% [0.0%, NaN%]	99.7% [99.2%, 99.9%]
ECI: Coagulopathy	13/1310	36.0% [26.1%, 47.3%]	10.4% [5.6%, 18.5%]	75.0% [65.6%, 82.5%]	68.5% [57.9%, 77.5%]	21.6% [13.9%, 32.0%]	95.9% [90.6%, 98.3%]
ECI: Obesity	101/1310	9.4% [6.2%, 14.1%]	17.5% [13.9%, 21.7%]	39.3% [26.3%, 54.1%]	96.9% [93.0%, 98.7%]	73.2% [52.3%, 87.1%]	88.3% [84.3%, 91.3%]
ECI: Weight loss	201/1310	11.2% [7.2%, 17.2%]	8.6% [4.6%, 15.6%]	44.7% [30.1%, 60.3%]	91.9% [88.1%, 94.6%]	34.1% [20.1%, 51.7%]	94.7% [90.2%, 97.2%]
ECI: Fluid and electrolyte disorders	30/1310	59.6% [51.0%, 67.7%]	11.5% [5.4%, 22.7%]	75.4% [69.2%, 80.7%]	42.4% [34.0%, 51.2%]	14.5% [7.1%, 27.5%]	93.0% [84.7%, 96.9%]
ECI: Blood loss anemia	35/1310	0.9% [0.4%, 2.0%]	6.7% [2.9%, 14.8%]	1.0% [0.1%, 10.4%]	99.1% [98.1%, 99.6%]	7.6% [0.6%, 54.1%]	93.3% [85.1%, 97.1%]
ECI: Deficiency anemia	54/1310	3.0% [1.4%, 6.2%]	12.1% [6.0%, 22.7%]	8.0% [1.7%, 30.0%]	97.7% [96.1%, 98.6%]	31.9% [7.3%, 73.7%]	88.5% [78.5%, 94.2%]
ECI: Alcohol abuse	21/1310	5.5% [3.3%, 9.0%]	8.2% [7.1%, 9.5%]	42.8% [27.9%, 59.2%]	97.9% [93.0%, 99.4%]	64.2% [34.4%, 86.0%]	95.0% [92.9%, 96.6%]
ECI: Drug abuse	10/1310	1.2% [0.6%, 2.3%]	2.4% [1.8%, 3.1%]	25.7% [14.5%, 41.2%]	99.4% [98.0%, 99.8%]	49.7% [22.8%, 76.8%]	98.2% [97.4%, 98.8%]
ECI: Psychoses	12/1310	1.0% [0.4%, 2.4%]	2.5% [1.3%, 4.9%]	32.6% [12.7%, 61.7%]	99.9% [99.1%, 100.0%]	85.6% [24.2%, 99.1%]	98.3% [96.2%, 99.2%]
ECI: Depression	14/1310	6.6% [4.0%, 10.7%]	7.6% [3.5%, 15.7%]	41.7% [31.1%, 53.0%]	96.3% [94.2%, 97.7%]	48.2% [26.5%, 70.6%]	95.2% [90.2%, 97.8%]
Asplenia (acquired or congenital)	2/1310	0.2% [0.0%, 2.1%]	0.5% [0.1%, 2.1%]	38.3% [0.5%, 98.8%]	100.0% [100.0%, 100.0%]	100.0% [NaN%, NaN%]	99.7% [98.6%, 99.9%]
Previous solid organ transplantation	1/1310	4.1% [2.3%, 7.2%]	2.9% [1.4%, 5.8%]	96.2% [82.7%, 99.3%]	98.6% [97.1%, 99.3%]	67.3% [41.5%, 85.6%]	99.9% [99.6%, 100.0%]
Chemotherapy	1/1310	8.9% [2.9%, 24.0%]	5.4% [2.4%, 11.6%]	76.8% [26.4%, 96.8%]	95.0% [86.0%, 98.3%]	46.8% [36.1%, 57.7%]	98.6% [94.8%, 99.6%]
Stroke treatment	1/1310	2.8% [1.9%, 4.1%]	3.5% [2.1%, 6.0%]	50.3% [34.5%, 66.0%]	99.0% [97.5%, 99.6%]	64.6% [31.7%, 87.8%]	98.2% [96.6%, 99.1%]

Accuracy was investigated for coding by ICD-10-codes or OPS-codes (procedure codes – *Operationen- und Prozedurenschlüssel*) in IAHD (inpatient administrative health data) for risk-factors compared to the reference-standards obtained by review of medical records. Analyses were conducted for cases with severe sepsis-1 according to chart review. Missing values are given for the numbers in the sample and result from lacking information in medical records to judge the presence of the respective risk-factor. Statistics are presented as proportions (%) along with their 95% confidence intervals in squared brackets adjusted for sampling weights and clustering. If there was overlap in definition between categories of Charlson comorbidity index (CCI) and Elixhauser comorbidity index (ECI), the categories of the ECI are presented.

7 Supplemental references

1. Fleischmann C, Thomas-Rueddel DO, Hartmann M, Hartog CS, Welte T, Heublein S, Dennler U, Reinhart K, (2016) Hospital Incidence and Mortality Rates of Sepsis An Analysis of Hospital Episode (DRG) Statistics in Germany From 2007 to 2013. Dtsch Arztebl Int 113: 159-166. https://doi.org/10.3238/arztebl.2016.0159
2. Fleischmann-Struzek C, Thomas-Rüddel DO, Schettler A, Schwarzkopf D, Stacke A, Seymour CW, Haas C, Dennler U, Reinhart K, (2018) Comparing the validity of different ICD coding abstraction strategies for sepsis case identification in German claims data. PLOS ONE 13: e0198847. https://doi.org/10.1371/journal.pone.0198847
3. Fleischmann-Struzek C, Mikolajetz A, Schwarzkopf D, Cohen J, Hartog CS, Pletz M, Gastmeier P, Reinhart K, (2018) Challenges in assessing the burden of sepsis and understanding the inequalities of sepsis outcomes between National Health Systems: secular trends in sepsis and infection incidence and mortality in Germany. Intensive Care Med 44: 1826-1835. https://doi.org/10.1007/s00134-018-5377-4
4. Gwet KL, (2008) Computing inter-rater reliability and its variance in the presence of high agreement. Br J Math Stat Psychol 61: 29-48. https://doi.org/10.1348/000711006x126600
5. Grouven U, Bender R, Ziegler A, Lange S, (2007) Der Kappa-Koeffizient. Dtsch Med Wochenschr 132 Suppl 1: e65-68. https://doi.org/10.1055/s-2007-959046
6. Lumley T (2021) survey: Analysis of Complex Survey Samples (version 4.0) [Computer software]. The Comprehensive R Archive Network, Available from: https://cran.r-project.org/web/packages/survey/index.html
7. Beaumont J-F, (2004) On the use of data collection process information for the treatment of unit nonresponse through weight adjustment. Survey Methodology 31: 227-331.
8. Therneau T, Atkinson B, Ripley B, (2019) rpart: Recursive Partitioning and Regression Trees. https://cran.r-project.org/web/packages/rpart/index.html. Accessed 12.12. 2021
9. Long JA (2020) jtools: Analysis and presentation of social scientific data (R package version 2.1.0) [Computer software]. The Comprehensive R Archive Network, Available from https://cran.r-project.org/package=jtools
10. Duong T (2021) ks: Kernel smoothing (R package version 1.13.3) [Computer software]. The Comprehensive R Archive Network, Available from https://CRAN.R-project.org/package=ks
11. Schwarzkopf D, Fleischmann-Struzek C, Schlattmann P, Dorow H, Ouart D, Edel A, Gonnert FA, Götz J, Gründling M, Heim M, Jaschinski U, Lindau S, Meybohm P, Putensen C, Sander M, Reinhart K, (2020) Validation study of German inpatient administrative health data for epidemiological surveillance and measurement of quality of care for sepsis: the OPTIMISE study protocol. BMJ Open 10: e035763. https://doi.org/10.1136/bmjopen-2019-035763
12. Statistisches Bundesamt (Destatis) (2017) Fallpauschalenbezogene Krankenhausstatistik (DRG-Statistik) Diagnosen, Prozeduren, Fallpauschalen und Case Mix der vollstationären Patientinnen und Patienten in Krankenhäusern.
